# Advanced Piezoelectric Materials, Devices, and Systems for Orthopedic Medicine

**DOI:** 10.1002/advs.202410400

**Published:** 2024-12-12

**Authors:** Jingkai Zhang, Chang Liu, Jun Li, Tao Yu, Jing Ruan, Fan Yang

**Affiliations:** ^1^ Department of Orthopaedics Shanghai Key Laboratory for Prevention and Treatment of Bone and Joint Diseases Shanghai Institute of Traumatology and Orthopaedics Ruijin Hospital Shanghai Jiao Tong University School of Medicine Shanghai 200025 China; ^2^ Department of Ophthalmology Shanghai Ninth People's Hospital Shanghai JiaoTong University School of Medicine Shanghai 200011 China; ^3^ Department of Materials Science and Engineering University of Wisconsin–Madison Madison WI 53706 USA; ^4^ Research Institute of Frontier Science Southwest Jiaotong University Chengdu Sichuan 610031 China

**Keywords:** bone regeneration, implantable and wearable devices, orthopedic medicine, piezoelectric materials

## Abstract

Harnessing the robust electromechanical couplings, piezoelectric materials not only enable efficient bio‐energy harvesting, physiological sensing and actuating but also open enormous opportunities for therapeutic treatments through surface polarization directly interacting with electroactive cells, tissues, and organs. Known for its highly oriented and hierarchical structure, collagen in natural bones produces local electrical signals to stimulate osteoblasts and promote bone formation, inspiring the application of piezoelectric materials in orthopedic medicine. Recent studies showed that piezoelectricity can impact microenvironments by regulating molecular sensors including ion channels, cytoskeletal elements, cell adhesion proteins, and other signaling pathways. This review thus focuses on discussing the pioneering applications of piezoelectricity in the diagnosis and treatment of orthopedic diseases, aiming to offer valuable insights for advancing next‐generation medical technologies. Beginning with an introduction to the principles of piezoelectricity and various piezoelectric materials, this review paper delves into the mechanisms through which piezoelectric materials accelerated osteogenesis. A comprehensive overview of piezoelectric materials, devices, and systems enhancing bone tissue repair, alleviating inflammation at infection sites, and monitoring bone health is then provided, respectively. Finally, the major challenges faced by applications of piezoelectricity in orthopedic conditions are thoroughly discussed, along with a critical outlook on future development trends.

## Graphical abstract

Harnessing the robust electromechanical coupling of piezoelectric materials, this review explores their applications in orthopedic medicine, inspired by collagen's natural piezoelectricity in bones. It covers how these materials accelerate osteogenesis, regulate cellular microenvironments through ion channels and signaling pathways, and enhance bone tissue repair, inflammation alleviation, and health monitoring. Finally, it addresses the challenges of integrating piezoelectricity into orthopedic treatments and provides insights into future development trends for next‐generation medical technologies.

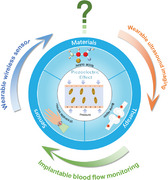

## 1 Introduction

Bone is a metabolically active, highly mineralized connective tissue essential for structure, support, movement, and microcirculation.^[^ ^1^, ^2^, ^3^ ^]^ The skeleton includes dense and cancellous bone, primarily comprising cells and a calcium‐rich extracellular matrix.^[^ ^4^, ^5^, ^6^, ^7^, ^8^, ^9^ ^]^ The structure features the central medulla (bone marrow cavity), bone tissue, and periosteum. Bone cells, such as mesenchymal stem cells, osteoblasts (OBs), and osteoclasts (OCs), are important. OBs mature into bone cells, secreting the matrix, while OCs handle bone resorption, maintaining metabolic balance.^[^ ^10^, ^11^, ^12^, ^13^, ^14^ ^]^ This balance can be disrupted by diseases, causing OCs to release destructive cytokines such as TRAP, (matrix metalloproteinase, MMP)‐9, and (cathepsin K) Ctsk, necessitating extensive osteoblast differentiation for bone repair.^[^ ^15^, ^16^ ^]^

The discovery piezoelectric properties in bone dates back to 1954, attributing to the shearing of collagen fibers.^[^ ^17^ ^]^ These piezoelectric, ferroelectric, and other bioelectric signals within natural living bone play crucial roles in regulating bone repair and growth. In dry bone, piezoelectricity primarily arises from the sliding of collagen fibers under mechanical forces during various bodily activities, inducing compression and other stresses on the bone.^[^ ^18^, ^19^ ^]^ This stress prompts collagen to exhibit piezoelectric properties, generating positive and negative charges under compression and tension, respectively.^[^ ^20^, ^21^, ^22^ ^]^ These negative charges can enhance osteoblast function and promote bone regeneration.^[^ ^18^ ^]^ The magnitude of piezoelectric energy produced within the bone largely depends on the direction of pressure applied to the bone axis. Notably, a high piezoelectric constant is observed when pressure is applied at a 45° angle to the bone axis.^[^ ^23^ ^]^ Moreover, studies indicate minimal variation in the piezoelectric coefficient across the human tibia, with the coefficient remaining relatively uniform throughout the bone structure.^[^ ^24^ ^]^ In wet bone, monitoring piezoelectric performance becomes challenging or may exhibit lower piezoelectric properties due to water molecules, which increase matrix conductivity and disrupt collagen's asymmetry, thereby diminishing piezoelectric performance.^[^ ^25^ ^]^ Overall, the endogenous electric field within living bone effectively regulates intracellular cell metabolism.^[^ ^20^ ^]^

Various injuries or diseases can result in bone tissue defects. For example, osteoporosis is a common disorder of bone metabolism, particularly prevalent in postmenopausal women, which predisposes them to fractures and other bone injuries.^[^ ^26^, ^27^, ^28^, ^29^ ^]^ Joint inflammation conditions such as osteoarthritis manifest with bone damage and persistent joint inflammation.^[^ ^30^, ^31^, ^32^ ^]^ These ailments and diverse bone injuries can lead to bone defects, requiring interventions to promote bone healing and regeneration. Currently, the prevailing methods for treating bone injuries involve prolonged fixation and bone transplantation.^[^ ^33^, ^34^ ^]^ Electrical stimulation has been a therapeutic approach since the 1980s, demonstrating efficacy in enhancing bone healing and regeneration. It works by using electric fields to stimulate osteoblast activity, promoting bone formation, and repair. Despite its effectiveness, conventional electrostimulator devices face significant drawbacks, including bulky designs, frequent battery replacements, and limited integration with the body.^[^ ^35^, ^36^, ^37^ ^]^ These limitations restrict their long‐term use and practicality in clinical settings.

Piezoelectric materials are known for their capabilities to convert mechanical energy into electrical energy, making them promising in biomedical engineering. Leveraging the piezoelectric effect, these materials engage in effective interfacial charge transfer interactions with cells and biological processes. The inherent electromechanical coupling present in bones allows producing local electricity that triggers osteoblast stimulation, facilitating bone formation. This process highlights the substantial potential of piezoelectric materials, including both inorganic and organic ones, in the field of orthopedic medicine.^[^ ^38^ ^]^

**Figure 1:**
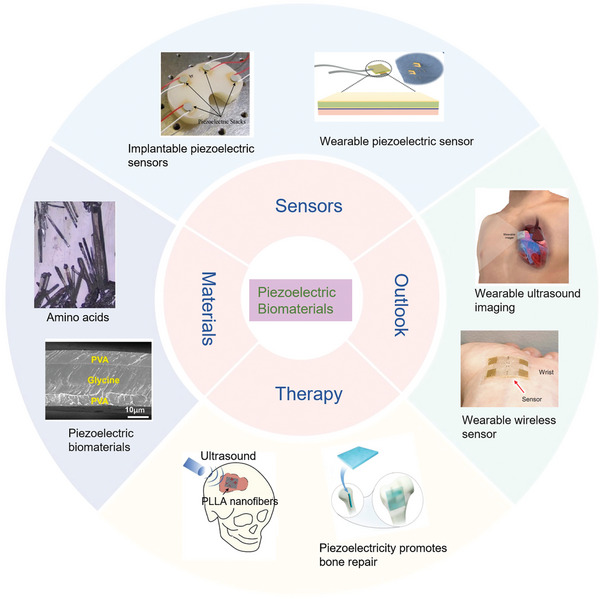
Piezoelectric materials and orthopedic medicine. Materials: Reproduced with Permission.^[^ ^43^ ^]^ Copyright 2020 Elsevier Ltd. Reproduced with Permission.^[^ ^83^ ^]^ Copyright 2021, American Association for the Advancement of Science. Therapy: Reproduced with Permission.^[^ ^184^ ^]^ Copyright 2020, Elsevier BV. Reproduced with Permission.^[^ ^186^ ^]^ Copyright 2020, Wiley. Sensors: Reproduced with Permission.^[^ ^229^ ^]^ Copyright 2018, 10P Publishing Ltd. Reproduced with Permission.^[^ ^261^ ^]^ Copyright 2019, American Chemical Society. Outlook: Reproduced with Permission.^[^ ^270^ ^]^ Copyright 2023, Nature Publishing Group. Reproduced with Permission.^[^ ^271^ ^]^ Copyright 2022, American Association for the Advancement of Science.

To explore novel medical technologies in orthopedics, this review introduces emerging applications of piezoelectric materials in the diagnosis and treatment of bone disorders. Initially, the review provides a general background on the principles of piezoelectric effect, the characteristics of various piezoelectric materials, and their preparation methods. It then discusses the working mechanisms of piezoelectric materials in bone injury repair. Furthermore, it overviews the medical applications of piezoelectric materials, devices, and systems in enhancing bone tissue repair, alleviating inflammation at infection sites, and monitoring bone health. Furthermore, the review discusses the primary challenges faced by piezoelectric materials in orthopedic medical applications and suggests direction for future studies, which include the development of new materials, seamlessly integration of biological and electrical functions, and the potential uses in personalized and precision medicine. Distinguished from other works discussing piezoelectric materials and biomedical applications,^[^ ^4^, ^12^, ^20^, ^34^, ^39^, ^40^ ^]^ our work comprehensively integrates fundamental physics principles, innovative material and device design, interacting mechanisms with complex biological systems, and advanced applications spanning from sensing to therapeutizing and to diagnosing, aiming to provide an in‐depth understanding of how piezoelectricity can be effectively utilized in various aspects of orthopedic conditions. Our review offers valuable insights into piezoelectric orthopedic technology, potentially guiding its advancement towards clinical translation and tailored therapeutic interventions as shown in **Figure** **1**.

## 2 Piezoelectricity and Piezoelectric Materials

**Figure 2:**
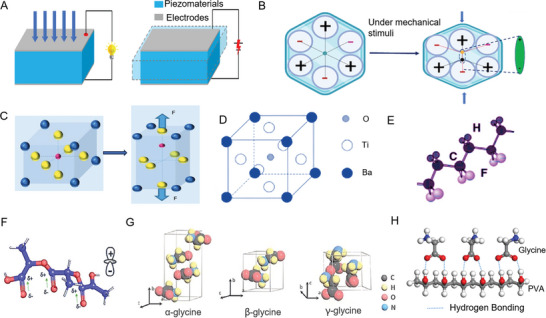
Piezoelectric materials and principles. A) Piezoelectric effect and reverse piezoelectric effect. B) Microscopic piezoelectric effect. Reproduced with Permission.^[^ ^49^ ^]^ Copyright 2022, MDPI. C) Molecular structure of lead zirconate titanate (PZT) inorganic piezoelectric materials. Reproduced with Permission.^[^ ^52^ ^]^ Copyright 2019, Wiley. D) Molecular structure of BaTiO_3_. Reproduced with Permission.^[^ ^67^ ^]^ Copyright 2022, MDPI. E) Polyvinylidene fluoride (PVDF) molecular structure formula. Reproduced with Permission.^[^ ^44^ ^]^ Copyright 2022, The Royal Society of Chemistry. F) Poly‐l‐lactic acid (PLLA) molecular structure formula. Reproduced with Permission.^[^ ^52^ ^]^ Copyright 2019, Wiley. G) Glycine molecular structure formula. Reproduced with Permission.^[^ ^72^ ^]^ Copyright 2022, John Wiley and Sons. H) Schematic diagram of PVA and glycine composite materials.^[^ ^83^ ^]^ Copyright 2021, American Association for the Advancement of Science.

The piezoelectric effect exhibits a linear relationship between mechanical stimulation and electrical response. In the absence of external force, the negative and positive charge centers within unit cell overlap, resulting in electrical neutrality.^[^ ^8^, ^41^ ^]^ Under external mechanical stress, the internal structure of unit cells deforms, leading to a separation of the charge centers and generating electric dipoles. This disturbance in the crystal structure and the shift in the polarization direction of the electric dipoles under mechanical force generates voltage. The extent of mechanical stress correlates directly with the degree of polarization change and the quantity of electrical energy produced, a phenomenon known as the direct piezoelectric effect^[^ ^42^, ^43^ ^]^ (**Figure** **2**A). Conversely, the material can generate mechanical motion through electrical energy, known as the inverse piezoelectric property^[^ ^44^, ^45^, ^46^, ^47^, ^48^, ^49^ ^]^ (Figure 2B). The piezoelectric coefficient, which represents the ratio between the induced strain and the difference in electric field strength, serves as a crucial metric for evaluating piezoelectric material performance. The variations in piezoelectric coefficients and properties across different materials are detailed in **Table** **1**. Historically, piezoelectric materials have been employed for centuries in applications such as force or pressure sensors, converters, and generators. Furthermore, these materials can be engineered into nanostructures and microstructures for monitoring biological forces and can replace passive conductors and capacitive polymers in self‐powered sensors.^[^ ^50^ ^]^

**Table 1:** Physical properties and medical applications of different piezoelectric materials.

Material properties	Piezoelectric materials/composites		Piezoelectric coefficient	Medical application	References
Inorganic	ZnO		*d*_33_: 12 pC N^−1^	Excellent transparency, high electron mobility, biocompatibility, and high processing temperature	[272]
	BaTiO_3_	10 nm BaTiO_3_ NPs	*d*_eff_: 0.45 pm V^−1^	Induce higher overall water splitting activity	[273]
		BaTiO_3_ thin film (Without poling) BaTiO_3_ thin film (Poled)	*d*_33_: 40 pm V^−1^ *d*_33_: 105 pm V^−1^	High voltage coefficient, good flexibility	[274]
		BaTiO_3_‐PHBV	/	Promote bone repair	[5]
		10wt%Ag‐TMSPM‐pBT	*d*_33_: 0.9 pC N^−1^	The piezoelectric constant close to bone tissue promotes bone repair	[179]
		BaTi0_3_/Ti6Al4V	/	Good biosafety and can promote bone repair	[13]
		(P(VDFTrFE)/BT)	/	Good biodegradability and can promote bone repair	[181]
		BT/PLA	*d*_33_: 7.03 ± 0.26 pC N^−1^	Good piezoelectricity and can promote bone repair	[85]
	PZT		*d*_33_: 40 pm V^−1^	High piezoelectric coefficient, Highly toxic lead	[60]
	KNN		*d*_33_: 90 pC N^−1^	High voltage electrical coefficient promotes bone repair	[275]
	AIN		*d*_33_: 4.76 pC N^−1^	Chemical stability, good electromechanical coupling ability	[276]
Natural biomolecules	Glycine	γ‐glycine	*d*_33_: 10.4 pm V^−1^	Good piezoelectricity but high brittleness	[277]
		PVA‐glycine‐PVA sandwich heterostructure	*d*_33_: 5.3 pC N^−1^	Excellent biodegradability and conductivity can be used for biosensors	[83]
		β‐ glycine	*d*_16_: 174 pm V^−1^	High piezoelectric coefficient but poor thermal stability	[278]
		β‐ Glycine/chitosan film	/	Due to its good piezoelectric output signal, it is applied in biocompatible piezoelectric sensors	[80]
	Diphenylalanine(FF)		*d*_33_: 17.9 pC N^−1^ *d*_15_: 60 pC N^−1^	Simple structure, high hardness, high high‐voltage electrical coefficient	[279]
		A 100 nm FF nanotube (NT)	*d*_15_: 35 pm V^−1^	Sensors with green nano piezoelectric materials that can come into direct contact with humans	[280]
		Aligned vertical FF microrods arrays	*d*_33_: 17.9 pm V^−1^	Good biocompatibility and piezoelectricity make it suitable for use in biomedical sensors	[281]
		Horizontally aligned FF NTs	*d*_15_: 46.6 pm V^−1^	Good biosafety and fast degradation rate can be used as biosensors	[282]
	Cyclo‐GW		*d*_16_: 14 pC N^−1^	Mechanical strength, high thermal stability, and high voltage coefficient	[272]
	Collagen		*d*_14_: −12 pm V^−1^	Biocompatibility, biodegradability, high flexibility, low toxicity, and ease of manufacturing	[68]
	Silk		*d*_33_: 38 pm V^−1^	Outstanding biocompatibility, biodegradability, and high flexibility	[68]
	M13 phage		*d*_eff_: 0.3 ± 0.03 pm V^−1^	Viruses with piezoelectric properties, easy to assemble	[283]
	M13 Phage film		*d*_33_: 11.2 ± 0.7 pm V^−1^	High voltage electrical coefficient may be applied to actuators and energy harvesters	[283]
	Cellulose		*d*_33_: 19.3 pm V^−1^	Flexibility, degradability, and stretchability	[68]
	Chitosan		*d*_33_: 15.56 pC N^−1^	Degradable and biocompatible	[284]
	chitin		*d*_eff_: 3.986 pm V^−1^	Synthetic counterparts have higher crystallinity and tensile strength	[68]
Polymers	PVDF		*d*_33_: −30 pC N^−1^	Natural softness and stretchability, good piezoelectric characteristics, negative *d*_33_ value, and inertness	[285]
	PLLA		d14: 10 pC N^−1^	Good piezoelectricity	[286]
	BT/PLA		*d*_33_: 7.03 ± 0.26 pC N^c^	Good biodegradability and can promote bone repair	[85]
	PCL/graphene scaffolds		/	Good biosafety and biodegradability, and can promote bone repair	[32]

The piezoelectric effect in inorganic materials primarily arises from ion displacement within their crystalline structures. Under mechanical stress, these materials exhibit a shift in their atomic configurations, altering the ion balance and inducing dipole moments, which is the direct piezoelectricity.^[^ ^51^, ^52^ ^]^ Commonly used piezoelectric materials include lead zirconate titanate (PZT) (Figure 2C), quartz, aluminum nitride (AlN), zinc oxide (ZnO), barium titanate (BaTiO_3_), lithium niobate (LiNbO_3_), and quartz.^[^ ^53^, ^54^ ^]^ ZnO, a hexagonal piezoelectric crystal, demonstrates unique properties as piezoelectric ceramic. It exhibits low voltage coefficients and room‐temperature ferroelectricity together with large surface‐to‐volume ratio during synthesis, particularly suitable for sensor applications.^[^ ^55^, ^56^, ^57^, ^58^ ^]^ In contrast, polycrystalline ceramics such as AlN and PZT consist of numerous randomly oriented grains. PZT, notable for its high voltage potential and ferroelectric properties, can be applied as an effective energy harvester. The inherent brittleness of PZT can be mitigated by compositing with other materials, enhancing its flexibility and piezoelectric performance, making it particularly useful in sensor applications.^[^ ^59^, ^60^, ^61^ ^]^ However, the use of lead in PZT raises significant environmental and health concerns due to its toxicity.^[^ ^55^, ^62^ ^]^ Conversely, lead‐free materials such as BaTiO_3_ and potassium sodium niobate (KNN) are explored for their high voltage coefficients and ferroelectric properties.^[^ ^63^, ^64^, ^65^, ^66^, ^67^ ^]^ (Figure 2D)

Organic materials typically feature with low crystal symmetry. The piezoelectric effect in these materials primarily results from the motion of dipoles within the bulk polymer matrix.^[^ ^68^, ^69^ ^]^ The reorientation of dipole molecules, induced by stretching or the application of high electric fields, facilitates the emergence of piezoelectric properties. Among organic piezoelectric materials, polyvinylidene fluoride (PVDF),^[^ ^44^ ^]^ glycine, poly‐l‐lactic acid (PLLA), and collagen are most notable ones. Specifically, PVDF and its copolymers, which require mechanical stretching and polarization to exhibit significant piezoelectric properties, are extensively studied due to their high voltage electrical characteristics, chemical resistance, thermal stability, and superior processability and mechanical properties (Figure 2E).^[^ ^43^, ^44^, ^70^, ^71^ ^]^ PVDF can exist in five piezoelectric crystal phases, with the β‐phase featuring dipoles arranged in parallel, thus providing a high dipole moment per crystal cell and the strongest piezoelectric properties.^[^ ^72^, ^73^ ^]^ To further enhance PVDF's performance, various copolymers have been developed, offering improved crystallinity and flexibility, making them more suitable for tissue engineering applications.^[^ ^74^, ^75^, ^76^ ^]^ PLLA, a biodegradable biofilm, while possessing lower piezoelectric properties compared to inorganic piezoelectric ceramics, showcases a large piezoelectric shear constant in its membrane form (Figure 2F).^[^ ^52^, ^77^, ^78^ ^]^ During the stretching process, the alignment of dipoles along the stretch direction enables the transformation from the α‐crystal form to the piezoelectric β‐form, and fiber electrospinning may also enhance PLLA's piezoelectric properties.^[^ ^43^, ^59^ ^]^ Amino acids, essential components of protein crystals, play crucial roles in biological systems. The structural variability among amino acids is primarily due to their side chains, with glycine manifesting in α, β, and γ crystal structures under various crystallization conditions. The α‐Glycine crystals exhibit high symmetry and lack piezoelectric properties, whereas β and γ glycine crystals, due to their asymmetric structures, possess piezoelectric properties, with β‐glycine having a piezoelectric charge coefficient comparable to traditional organic piezoelectric materials (Figure 2G).^[^ ^72^, ^79^, ^80^ ^]^ Collagen, composed of a triple helix structure of three intertwined peptide chains, exhibits piezoelectric properties due to its asymmetric spatial structure and the abundance of polar and charged groups in its main chain. External mechanical stress leads to the reorientation of dipole moments in these amino acids along the longitudinal direction, resulting in changes in polarization and thus a piezoelectric response.^[^ ^72^ ^]^ Elastin, extensively studied and found in organs such as the skin and blood vessels, not only exhibits piezoelectric properties but also ferroelectric properties, which can be further enhanced through electroporation.^[^ ^81^ ^]^

Organic piezoelectric materials offer notable advantages, such as exceptional flexibility, high biocompatibility, and environmental friendliness, compared to inorganic counterparts. However, improvements are still needed in mechanical, piezoelectric, and biocompatibility performance. Organic composite piezoelectric materials have gained substantial attentions in research due to their lightweight nature, soft mechanical properties, low‐temperature processing, and excellent piezoelectric performance.^[^ ^82^ ^]^ For example, mixed membranes of glycine and PVA exhibit a stable piezoelectric coefficient, excellent degradability, and dissolve in water within 5 min, demonstrating both in vitro and in vivo biological safety (Figure 2H).^[^ ^83^ ^]^

Inorganic materials such as AlN, PZT, ZnO, and LiNbO₃ offer high piezoelectric coefficients (typically ranging from 12 to 90 pC N^−1^ according to Table 1) and broad responses but are limited by their hardness, brittleness, and poor biocompatibility.^[^ ^52^, ^84^, ^85^ ^]^ In contrast, organic piezoelectric materials such as glycine, collagen, and PLLA excel in flexibility, biocompatibility, and environmental sustainability, although their piezoelectric coefficients are generally lower (typically ranging from 5.3 to 60 pC N^−1^ according to Table 1). To address these challenges, hybrid composites combining inorganic and organic components are being developed. These composites aim to leverage the strengths of both material types, enhancing mechanical performance, biocompatibility, and piezoelectric output.^[^ ^83^, ^86^, ^87^, ^88^, ^89^ ^]^ As summarized in Table 1, these hybrid materials offer a balanced solution, making them well‐suited for a range of biomedical applications. For example, PVDF/BaTiO₃ composites exhibit improved performance, increasing the *d*₃₃ coefficient from 0.7 to 2.6 pC N^−1^.^[^ ^90^ ^]^ Incorporating graphene oxide (GO) into PVDF through electrospinning promotes β‐phase formation, quadrupling the piezoelectricity compared to pure PVDF.^[^ ^91^ ^]^ Embedding ZnO particles in PVDF via laminar flow‐assisted electrospinning forms iron electret fibers, achieving *d*₃₃ values of 56 ± 2 pC N^−1^, significantly surpassing typical polymer‐based materials.^[^ ^92^ ^]^ Further innovations include the integration of Fe₃O₄ magnetic nanoparticles (NPs) into polyacrylonitrile (PAN) fibers through magnetically assisted electrospinning. This method enables a stable *d*₃₃ value of 4.5 pm V^−1^ while addressing the need for a 3D cell‐growth environment.^[^ ^93^ ^]^ Similarly, PVDF‐HFP polymers with Li‐KNN microparticles achieve high conductivity (6.34 S m^−1^) with enhanced particle‐matrix compatibility through surface modifications.^[^ ^94^ ^]^ KNN particles embedded in PVDF matrices have also been used to fabricate ferroelectric arteries, which can be rapidly polarized during printing. These arteries exhibit excellent pressure sensitivity, with a *d*₃₃ value of 12 pC N^−1^, enabling real‐time monitoring of blood pressure and vasomotor patterns for early detection of vascular blockages.^[^ ^95^ ^]^

In tissue repair, it is crucial to ensure the elastic modulus of the selected biomaterial aligns with the target tissue. Bone, as a heterogeneous, multiphase composite material, exhibits variable elastic modulus (Young's modulus) due to differences in microstructure. For example, the same bone may display an elastic modulus ranging from 4 to 34 GPa depending on humidity and testing conditions.^[^ ^96^ ^]^ Compared to inorganic conductive materials, 3D polymer scaffolds provide greater flexibility, reduced stiffness, and lower Young's modulus, making them more suitable for softer tissues like the nervous system.^[^ ^97^ ^]^ While a higher modulus can hinder wound healing, lowering material stiffness has been shown to facilitate tissue recovery.^[^ ^98^ ^]^ For instance, the compression modulus of mandibular trabeculae ranges from 24.9 to 240 MPa, with an average of 96.2 ± 40.6 MPa, whereas cancellous bone varies between 50 and 500 MPa, depending on bone quality. In vitro tests of scaffolds with a compression modulus of 244 ± 16 MPa confirmed excellent biosafety, along with strong cell adhesion and growth.^[^ ^78^ ^]^ Besides, a 3D printed artificial bone structure with bone‐like mechanical properties and ceramic‐like piezoelectricity was also reported. Additionally, by changing the porosity and component ratio, the modulus and fracture toughness of the 3D printed artificial bone can be tuned to exactly match the mechanical properties of cancellous bones, providing a solution for seamless integration with orthopedic system.^[^ ^94^ ^]^

Enhancing the biological performance of piezoelectric materials is crucial for promoting tissue regeneration. Several forms of advanced material have been explored in biomedical research, including fibers, membranes, films, scaffolds, microcapsules, hydrogels, and composites. Each form offers unique advantages depending on the application. Composites, such as piezoelectric bioactive glasses composite (P‐KNN/BG), mimic the bone microenvironment and promote angiogenesis through radiostimulation and sustained ion release, improving endothelial cell adhesion and migration.^[^ ^99^ ^]^ Electrospun nanofibers, such as aspalathin‐loaded P(VDF‐TrFE)‐Eudragit 1100‐AuNP fibers, enhance hydrophilicity, reduce fiber diameter, and improve piezoelectricity through the incorporation of gold NPs.^[^ ^100^ ^]^ Microcapsules, immobilized on PCL, PHB, and PHB‐PANi scaffolds, provide controlled drug release in response to physical or biological stimuli, demonstrating effective dexamethasone release in vitro.^[^ ^101^ ^]^ Core–shell nanofibers made from PLLA/glycine exhibit excellent β‐phase alignment and surface adaptability, enabling precise detection of physiological movements, making them ideal for sensors in biological tissues.^[^ ^102^ ^]^ KNN‐based spatio‐temporal microcapsules generate reactive oxygen species (ROS) under ultrasound (US) stimulation, eliminating bacteria and gradually releasing vascular endothelial growth factor (VEGF) to promote angiogenesis and wound healing.^[^ ^103^ ^]^ Similarly, E‐MoS₂/PVDF microcapsules utilize bubble‐driven piezocatalysis to enhance dissolved oxygen levels and accelerate antibiotic degradation through piezoelectric catalysis.^[^ ^104^ ^]^

## 3 Biological Mechanisms of Accelerated Osteogenesis by Piezoelectrical Materials

**Figure 3:**
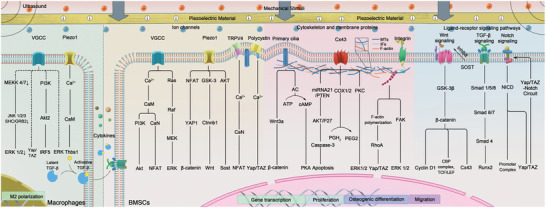
Mechanisms of accelerated osteogenesis by piezoelectric stimulus. The dashed lines marked the potential electrosensitive transducer.

Piezoelectric materials benefit osteogenesis and mineralization through mechanical powder‐induced electrical stimuli. In the meanwhile, the single mechanical stress also could promote osteoblasts effects in maintaining homeostasis of bone metabolism. Piezoelectric stimulation‐induced osteoconductivity in stem cells primarily depends on voltage‐ and mechanosensitive ion channels, cytoskeletal proteins, membrane proteins, and ligand–receptor signaling pathways (**Figure** **3**, **Table** **2**).

**Table 2:** Effects of electrical stimulation on cellular signaling pathways.

Molecular sensor type	Signaling pathway	Model	Stimuli type	Stimuli factor	Reference
Ion channel	Voltage‐gated ion channel/Ca^2+^‐Calmodulin	BMSCs	Indirect, electrical	Electrospun Hap composited PVDF‐TrFE nanofiber	[106]
		hMSCs/Mouse calvaria (In vivo)	Indirect, electrical	HAp nanoparticle composited PVDF‐TrFE	[287]
		MC3T3‐E1	Direct, electrical	Coupled electric field (60 Hz, 20 mV cm^−1^)	[288]
		MC3T3‐E1	Direct, electrical	Coupled electric field (60 Hz, 20 mV cm^−1^)	[289]
	Trpv1/Calcium influx/cAMP signaling	hMSCs	Mechanical	1‐Pa oscillating fluid shear applied through a 10 ml syringe (flow 52.5 mL min^−1^, frequency 1 Hz)	[123]
		MLO‐Y4 osteocyte‐like cells	Mechanical	1‐Pa peak shear stress on the slide surface (peak flow rate: 18.8 mL min^−1^; chamber dimensions: 56 × 24 × 0.28 mm)	[119]
	Smad/BMP/IGF1 signaling	MC3T3‐E1	Direct, electrical	Electrical field applied using a function signal generator (square wave, 100 Hz frequency, 200 × 10^−9^ m voltage, duty ratio 50%)	[166]
	polycystin‐1/YAP/TAZ signaling	C3H10T1/2 (ATCC) cells	Mechanical	Cyclical stretch (10%, 0.5 s of stretch and 0.5 s of relaxion, 15 cycles per minute)	[290]
	Piezo1/YAP/TAZ signaling	Murine tibial (In vivo)	Mechanical	Physiological mechanical loading deprivation (tail suspension)	[133]
	Piezo1/Calcium influx	MG‐63 cell/Rat femur (In vivo)	Indirect, electrical	PMMA/PEI/PVDF implantation	[291]
		RAW264.7/BMSCs	Mechanical	Cyclic sinusoidal continuous tensile strain for 0, 1, 2, 4 and 6 h (10%, 0.5 Hz)	[170]
		RAW264.7/MSCs/Murine tibial (In vivo)	Mechanical	Continuous 2‐Hz sinusoidal waveform ranging from 2‐N compressive loading and a 12‐N peak loading for 360 cycles per day on three consecutive days per week	[134]
		BMSCs/human umbilical vein endothelial cells (HUVECs)	Mechanical	Pulsed triboelectric stimulation applied by wearable pulsed triboelectric nanogenerator	[292]
	Piezo1, microRNA‐29a, Wnt signaling	Ovariectomized mice (In vivo)	Mechanical	Pulsed piezoelectric microvibration (60, 120, and 180 Hz)	[293]
	Calcineurin/NFAT	MC3T3‐E1 cell	Indirect, electrical	Electroactive biocomposite of poly(lactic‐co‐glycolic acid) mixed with gadolinium‐doped barium titanate nanoparticles (Gd‐BTO NPs)	[110]
	Voltage‐gated ion channel/Ca^2+^‐ERK1/2 signaling	MYO‐Y4 cell	Mechanical	Hypotonicity induced membrane stretching (medium deluted by equal volume ddH2O)	[118]
Cytoskeletal and adhesion related protein	Primary cilia/COX‐2 signaling	MLO‐A5 mature osteoblast cells/Murine ulnar (In vivo)	Mechanical	Axial compressive load (3 N, 2 Hz sine wave for 120 cycles per day for 3 consecutive days)	[294]
	Integrin/Wnt/β‐catenin signaling	BMSCs	Mechanical	Enhanced extracellular matrix stiffness	[146]
	integrin/Gap junction Connexin 43	MLO‐Y4 osteocytic cells	Mechanical	Tibial mechanical loading (2 Hz haversine waveform for 600 cycles; 8.86 or 7.44 N)	[295]
	Integrin signaling	Murine tibial (In vivo)	Mechanical	Axial compressive load (triangular waveform, 4 ​Hz, 9.0 ​N peak force, 1200 cycles per loading)	[145]
	Gap junction Connexin 43/PEG2	MC3T3‐E1	Mechanical	A shear stress of 20 dyn cm^−2^ applied by an 18 mL min^−1^ flow rate	[296]
	Gap junction Connexin 43/ERK1/2 MAPK signaling	Osteocyte‐like MLOY‐4 cell	Mechanical	Cells were exposed for 1 h to oscillating fluid flow at a shear stress of ±10 dyn cm^−2^ and a frequency of 1 Hz in a parallel plate flow chamber	[151]
	Gap junction Connexin 43/Ca^2+^‐Calmodulin	MSCs	Direct, electrical	TiO_2_ implantation (0.4 V cm^−1^ external electric field performed)	[153]
		Mice tibial (In vivo)	Mechanical	Physiological mechanical loading deprivation (tail suspension)	[297]
		Osteocyte‐like MLOY‐4 cell	Mechanical	Steady laminar flow of 4, 8, and 16 dynes cm^−2^; pulsating fluid flow at 16 ± 0.8 dynes cm^−2^ at a frequency of 5 Hz	[298]
		ROS 17/2.8 cell	Mechanical	Cyclic stretch for 2 or 24 h less than 160,000 microstrain (µE) at the edge less than 20 000 µE at the center of the well	[299]
	Focal adhesions (FAs), focal adhesion kinase (FAK) and associated mechanotransduction signaling axis	MC3T3‐E1	Indirect, electrical	Ultrasound activated piezoelectric BaTiO_3_/TC4 material	[300]
	primary cilia/COX‐2 signaling	MC3T3‐E1	Mechanical	35 Hz, a cell surface stress magnitude of 0.25 g, 20 min day^−1^	[301]
	Primary cilia/MAPK/ERK signaling	Bovine articular chondrocytes	Mechanical	Sinusoidal US at 5.0‐MHz, pressure amplitude of either 14 or 60 kPa, 4 × 5‐min or 4 × 20‐min per day	[302]
Other physical responsive signaling pathway	Ca^2+^/Smad 1/5/8 signaling	BMSCs/RAW264.7	Indirect, electrical	BaTiO_3_/β‐TCP (BTCP) ceramic	[84]
	Wnt‐β‐catenin signaling	Mice ulna (In vivo)	Mechanical	Axial pulsed compressive loading (2 Hz, 1 min day^−1^)	[162]
		Murine BMSCs	Magnetic‐field‐induced surface potential	CoFe2O4 (CFO)/TbxDy1‐xFe2 alloy (TD)/P(VDF‐TrFE) Magnetoelectric response film	[303]
	TGF‐β signaling	Human chondrocytes SV40	Mechanical	Shear stress of 6.58 dynes cm^−2^ is achieved at a rotating frequency of 118 rpm.	[304]
		Bovine articular cartilage cells	Mechanical	3.5 Pa fluid shear stress (FSS) for 96 h.	[168]
		rBMSCs	Direct, electrical	Solid and nanoporous micropyramid patterned Si biomaterial, electrical field applied (1.5 V cm^−1^, 1 Hz)	[167]
	PI3K/Akt signaling	HUVECs/MC3T3	Indirect, electrical	Porous piezoelectric hydrogel bone scaffold fabricated by incorporating polydopamine (PDA)‐modified ceramic hydroxyapatite (PDA‐hydroxyapatite, PHA) and PDA‐modified barium titanate (PDA‐BaTiO_3_, PBT) nanoparticles (NPs) into a chitosan/gelatin (Cs/Gel) matrix.	[305]
		BMSCs/Rat calvaria (In vivo)	Indirect, electrical	Polydopamine‐modified hydroxyapatite (PHA), and barium titanate (PBT) coated PHBV biomimetic periosteum	[5]
		Rat macrophage/Rat femur (In vivo)	Indirect, electrical	Electrospun TiO_2_ nanotube (NT)/PVDF implantation	[306]
	BMP2/Smad signaling	HUVECs/MSCs/Rat calvaria (In vivo)	Indirect, electrical	Gold nanodots decorated rGO‐Hydroxyapatite nanocomposites piezoelectric bone cements	[307]
	Notch signaling	BMSCs/Murine tibial (In vivo)	Mechanical	In vivo cyclic compressive loading (216 cycles at 4 Hz, peak strains at a tibial midshaft of +900 µε)	[158]
		Human periodontal ligament cells	Mechanical	Intermittent compressive force (0.23 Hz at a 1.5 g cm^−2^ force)	[308]
	eNOS/NO signaling	HUVECs	Indirect, electrical	Polarized potassium sodium niobate composited piezoelectric bioactive glasses	[309]
	Notch/MAPK/SMAD signaling	hBMSCs	Direct, electrical	Gelatin‐polypyrrole hydrogel, electrical field applied (250 mV/20 min day^−1^)	[310]

### 3.1 Voltage‐ and Mechanosensitive Ion Channels Facilitate Osteogenesis

The voltage‐ and mechanosensitive ion channels are sensitive to electrical stimulation and mechanotransduction through ion channels protein. Voltage‐gated calcium channels (VGCCs), a classical transmembrane protein, is responsible for mediate intracellular Ca^2+^ influx under membrane potential changes.^[^ ^105^ ^]^ As a crucial intracellular second messenger, Ca^2+^ plays a significant role in stem cells osteogenic differentiation and osteogenesis.

Different members of VGCC, including Cav1.2 (a member of the L‐type VGCC family) and Cav3.1 (a member of the T‐type VGCC family) have been reported to be upregulated when subjected to electric stimulation and facilitate osteoblast mineralization and proliferation through the Wnt/β‐catenin, PI3K‐Akt and calcineurin/nuclear factor of activated T‐cell (NFAT) signal pathways.^[^ ^106^, ^107^, ^108^, ^109^, ^110^ ^]^ The terminal events of electrical stimulation signaling are the enhanced expression of osteogenic‐related proteins such as osterix (OSX), Runt‐related transcription factor 2 (RUNX2), alkaline phosphatase (ALP), osteopontin (OPN), and osteocalcin (OCN), although the upstream signaling pathways are complex.^[^ ^111^, ^112^, ^113^, ^114^ ^]^ Interestingly, electronegativity also influences cells fate. Negatively charged piezoelectric materials surfaces could induce Ca^2+^ influx and improve BMSC osteogenetic differentiation, while positively charged piezoelectric materials surfaces could promote M1‐like macrophage polarization to M2‐like macrophage, which is beneficial for tissue regeneration.^[^ ^84^ ^]^ Numerous studies have demonstrated that electrical stimulation can regulate bone homeostasis by influencing macrophage migration and polarization. For instance, electric fields have been shown to directly modulate macrophage migration while simultaneously upregulating the expression of PI3K and ERK.^[^ ^115^ ^]^ Electric stimulation could promote M2‐like macrophage polarization and benefit to the bone regeneration through regulating YAP/TAZ, PI3K/Akt, and MAPK signaling pathways.^[^ ^13^, ^116^, ^117^ ^]^ But whether VGCC act as a signaling transducer is still underlying. Finally, VGCC is also reported to sensitive to swelling‐induced mechanical strain, studies in MLO‐Y4 osteocyte‐like cells demonstrated that mechanical loading could activate Cav 3.2, followed by upregulation of phosphorylated ERK and ATP release.^[^ ^118^ ^]^

Primary cilia is an essential cellular mechanical sensor that mediates intercellular Ca^2+^ release and subsequent osteogenesis.^[^ ^119^ ^]^ In vitro experiment performed on MSCs demonstrated that the primary cilia is sensitive to surface topography and regulates osteogenesis mediated by Wnt3a/β‐catenin signaling.^[^ ^120^ ^]^ A mechanosensing mechanism independent of intracellular Ca^2+^ release is also reported that MLO‐Y4 cells respond to fluid shear stress (FSS) through activation of adenylyl cyclase6 (AC6) and consequently increased cAMP production.^[^ ^119^ ^]^ The primary cilia mechanosensitivity relies on transient receptor potential (TRP) family members, such as TRPV4 and polycystins. TRPV4 is indispensable for MSCs mechanical sensing and osteogenic differentiation,^[^ ^121^ ^]^ when subjected to mechanical stress, TRPV4‐mediated calcium influx is activated,^[^ ^122^, ^123^ ^]^ followed by the activation of downstream calcineurin/NFAT signaling pathway.^[^ ^124^ ^]^ Polycystins is another class of mechanically sensitive protein, which belongs to the TRP family member. Ion channel complexes consisting of polycystins are located on the cell surface and cilia. A mechanotransduction signaling complex is reported, consisting of PC1 and Wwtr1, mediates the mechanical loading responses of osteoblasts.^[^ ^125^ ^]^ The interaction between polycystins and TAZ can activate osteogenesis.^[^ ^126^ ^]^

Piezo1, a mechanically sensitive calcium‐permeable channel, is a nonselective cation‐conducting channel with a slight preference for Ca^2+^.^[^ ^127^, ^128^, ^129^ ^]^ Piezo1 is sensitive to mechanical stimuli, including FSS and extracellular matrix stiffness, and subsequently activates Ca^2+^ influx to stimulate calcineurin, followed by the dephosphorylation of NFATc1, YAP1, and β‐catenin, as well as NFAT/YAP1/β‐catenin complex formation.^[^ ^130^ ^]^ These activated transcription factors are indispensable for bone formation and mineralization. In contrast, the absence of Piezo1 induces severe bone loss in newborn mice and exhibits small response to the load, because of the specific activation of GSK3 which promotes Ctnnb1 degradation and then mitigate bone mass loss.^[^ ^131^ ^]^ Recent research shows that Piezo1 mediates force induced bone formation by activating Akt and then attenuating the express of sclerostin (SOST).^[^ ^132^ ^]^ Furthermore, Piezo1 regulates bone homeostasis through influence osteoclast function. Mice with Piezo1‐deficient osteoclasts exhibit increased osteoclast activity, while conditional knockout of Piezo1 in osteoblasts leads to osteoporosis through impairing Col2α1 and Col9α production.^[^ ^133^ ^]^ Periosteum macrophages is also responsive to the Piezo1 and participate in the bone formation. Mechanical loading promotes the accumulation of M2 (CD68^+^F4/80^+^) macrophages in the periosteum via Piezo1 signaling, capable of expressing and activating TGF‐β1 to enhance osteogenesis.^[^ ^134^ ^]^

### 3.2 Cytoskeleton and Membrane Proteins Mediate Osteogenesis

Cytoskeleton is an important media to transduce mechanical cues into biological changes. Mechanical stimulation causes pressurization of interstitial fluid, leading to FSS sensed by cytoskeleton and associated proteins,^[^ ^135^ ^]^ and ultimately initiates mechano‐to‐chemo signaling events through signaling proteins such as ion channels, enzymes, and kinases. There are three kinds of cytoskeletal polymer: actin filaments (F‐actin), microtubules (MTs), and intermediate filaments (IFs), and therefore, understanding how cytoskeletal components sense and transduce mechanical signals could reveal the bone formation biological mechanism. F‐actin plays a pivotal role in maintaining bone homeostasis, while integrin‐linked kinase (ILK) could regulate actin expression through interacting with integrin. ILK deficiency could cause F‐actin disorganization and subsequently reduce bone formation and mineralization.^[^ ^136^ ^]^ After FSS stimulation, actin stress fibers rearrange forming dense stress fibers oriented parallel to the direction of FSS, which is essential for the activation of downstream signaling events to respond to FSS.^[^ ^137^, ^138^ ^]^ MTs are crucial in the response to FSS, involving calcium flux and the modulation of sclerostin expression. Recent study identified a mechanical stress responsive cascade, where FSS activates NOX2, generating ROS that trigger the opening of TRPV4 channels, leading to Ca^2+^ influx. The NOX2‐ROS elicited Ca^2+^ influx activate downstream kinase CaMKII, decreasing the abundance of sclerostin protein to adapt to the mechanical environment. In this event, the mechanoresponsive range of osteocytes to FSS is defined by the abundance of detyrosinated tubulin.^[^ ^139^ ^]^ IFs are an important component of the cytoskeleton, serving as scaffold for cytoskeleton and direct MT assembly to regulate several gene expression and cellular functions, such as motility and migration.^[^ ^137^ ^]^ Though the role of IFs in bone mechanotransduction is still poorly studied. Synemin, a type if IV IF protein functioning as an A‐kinase anchoring protein, mediates the phosphorylation of adjacent proteins, and regulates osteogenesis and bone formation.^[^ ^140^, ^141^ ^]^ Osteoblasts isolated from Synemin‐deficient mice exhibited increased expression of osteogenic genes, including Runx2 and osteocalcin, but, a low expression level of cyclin D1 leads to osteoblast loss in vivo.^[^ ^140^ ^]^

Cytoskeleton integrates with mechanical stimulus mainly depending on the focal adhesion (FA) complex, which could create a physical linkage between the cytoskeleton and the ECM through integrins. Integrins are heterodimeric transmembrane receptors that essential for cell to respond to mechanical environment change. When stimulated by mechanical stress, following F‐actin cytoskeleton polymerization, YAP/TAZ coordinating signals from Rho GTPase is activated by FA complex and finally promote osteogenesis and bone remodeling.^[^ ^142^ ^]^ To date, 24 αβ heterodimeric integrin family members have been identified: αυβ3 integrin is closely linked to osteoclastogenesis and osteoclastic resorptive activity, whereas αvβ3 and β1 integrins are predominantly expressed in osteocytes.^[^ ^143^ ^]^ Integrin interacts with Yap/TAZ and the loss of β1 and β3 integrins in osteocytes significantly impairs force‐responsive bone formation and induces bone mass loss and osteoporosis.^[^ ^144^, ^145^ ^]^ Integrin is responsible to ECM stiffness. Stiff ECM is capable of activating integrin/focal adhesion kinase (FAK) pathway and then upregulating ERK 1/2 phosphorylation levels.^[^ ^146^ ^]^ Cells' response to electrical stimuli (ES) also depends on integrin function.^[^ ^147^ ^]^ Upregulation of integrin α5β1 is observed in BMSCs when exposed in ES, leading to improved cell adhesion and osteogenic differentiation.^[^ ^84^ ^]^ A specialized mechanotransduction complex, comprising the purinergic channel pannexin1, the ATP‐gated purinergic receptor P2×7R, and the transiently open low‐voltage T‐type calcium channel Cav3.2, has been identified within the β3 integrin attachment foci on osteocyte processes, highlighting the unique mechanosensation and transduction capabilities of integrins.^[^ ^148^ ^]^

Connexin43 (Cx43), an integral membrane protein involved in forming gap junctions and hemichannels, facilitates the exchange of molecules under 1 kDa.^[^ ^149^ ^]^ Aging Cx43‐deficient MLO‐Y4 osteocytic (Cx43def) cells release much osteoclastogenic cytokines, including receptor activator of NFκB ligand (RANKL) and high‐mobility group box‐1 (HMGB1). They exhibit low pro‐survival microRNA miR21 expression, in level and then induce phosphatase and tensin homolog (PTEN) increasing and phosphorylated Akt reduction.^[^ ^150^ ^]^ Cx43 protein expression levels increase significantly within 30 min to 4 h postexposure to fluid flow treatment. Osteocytic MLO‐Y4 cells stimulated with oscillating fluid flow exhibit high phosphoserine content of Cx43 compared to control cells, and the flow‐induced phosphoserine is mediated by extracellular signal‐regulated kinase (ERK1/2), leading to enhanced gap junctional intercellular communication.^[^ ^151^ ^]^ Prostaglandin E2 (PEG2) is indispensable for Cx43 to mediate fluid flow induced osteogenic differentiation, the osteogenesis promoting effect of FSS could be partially blocked by cyclooxygenase inhibitor, which damps cellular PGE2 synthesis.^[^ ^142^, ^152^ ^]^ Cx43 is also sensitive to ES, mediating continuous calcium influx via Cx43 and thereby enhancing osteogenesis.^[^ ^153^ ^]^ Upregulated Cx43 expression can be detected in myoblasts and cardiomyocytes when incubated on piezoelectric materials.^[^ ^154^, ^155^ ^]^

### 3.3 Ligand–Receptor Signaling Pathways Benefit Cells Cross‐talk and Osteogenesis

The ligand–receptor signaling pathways involved in bone formation in response to piezoelectric materials include TGF‐β, Wnt, and Notch‐mediated signal pathways. The Notch pathway, a system responsive to mechanical stress, facilitates communication between adjacent cells. When the Notch receptor interacts with a ligand presented by a neighboring cell, it is subjected to a mechanical pull, resulting in the proteolytic cleavage of the Notch extracellular domain (NECD) and the release of the Notch intracellular domain (NICD). The NICD translocates to the nucleus, interacts with transcription factors, and drives gene transcription, subsequently regulating stem cell differentiation and self‐renewal.^[^ ^156^, ^157^ ^]^ Notch, serving as a mechanical sensor, is crucial for force‐induced osteogenesis. Notch inhibitors could reduce stretching stimulated osteogenesis through cytoskeletal modulation, and the absence of Notch2 has a more significant impact on osteogenesis process than Notch1.^[^ ^158^ ^]^ Tensile stress can activate the expression of Notch1 and Delta‐like ligand 4 (DLL4) in bone marrow endothelial cells (BMECs), where the NICD enhances YAP/TAZ activity, establishing a YAP/TAZ‐Notch circuit and secreting exosomes enriched with YAP, TAZ, Notch1, and DLL4, which promotes bone formation.^[^ ^159^ ^]^

Wnt, a secreted glycoprotein with an approximate molecular weight of 40 kDa, is involved in various physiological and pathological processes.^[^ ^160^ ^]^ Wnt stimulation suppresses GSK‐3β activity and induces the cytoplasmic accumulation of β‐catenin, which translocates to the nucleus and activates the expression of target genes with the T‐cell factor (TCF)/lymphocyte enhancer factor 1 (LEF1) and CREB‐binding protein (CBP) complex.^[^ ^161^ ^]^ The canonical Wnt signaling pathway is pivotal in regulating osteogenesis, promoting the differentiation of stem cells into mature osteoblasts and responding to both mechanical and electrical stimuli. Overexpression of SOST could inhibit the Wnt pathway induced by compressive loading. Axial compressive loading in transgenic mice with high SOST expression levels resulted in significantly reduced Wnt expression and bone formation.^[^ ^162^ ^]^ Combination of bioceramic scaffold and ES was applied to treat bone defect in rats to investigate their effect on bone tissue formation, and the rats receiving treatment demonstrated higher Wnt1 and β‐catenin levels, suggesting that Wnt signal is sensitive to ES.^[^ ^163^ ^]^ As the downstream from Wnt signaling, Cx43^[^ ^164^ ^]^ contribute to Wnt mediated force‐induced osteogenesis.

The transforming growth factor‐beta (TGF‐β) superfamily comprises over 40 members, including TGF‐βs, Nodal, Activin, bone morphogenetic proteins (BMPs), etc. TGF‐β signaling transmits across the cell membrane mediated by complexes of specific type I and type II serine/threonine kinase receptors. The TGF‐β signaling pathway plays a central role in the osteogenesis of stem cells and bone formation, influenced by both mechanical and electrical stimuli.^[^ ^165^ ^]^ The electrical field could directly regulate the TGF‐β signaling pathway, and electrical stimuli synergize with BMP‐2 to enhance osteogenesis via regulating the Smad signaling pathway,^[^ ^166^, ^167^ ^]^ which activates RUNX2 and then promotes bone formation.^[^ ^168^ ^]^ Mechanical loading can enhance the endochondral ossification through promoting BMP‐2 and TGF‐β1 combined application effect.^[^ ^169^ ^]^ Additionally, mechanical forces could indirectly regulate the TGF‐β pathway via macrophages.^[^ ^170^ ^]^ Briefly, macrophages can detect mechanical loading through the Piezo1 ion channel, leading to the activation of TGF‐β1 through the expression and secretion of thrombospondin‐1 (Thbs1). Inhibition of Thbs1 disrupts loading‐induced bone formation mediated by TGF‐β and periosteal osteoprogenitor assembly.^[^ ^134^ ^]^

Mechano‐and electrosensitive ion channels, the cytoskeleton and associated membrane proteins, along with ligand–receptor signaling pathways, constitute a complex regulatory signaling network, which plays a vital role in osteogenesis response to piezoelectric materials under the external mechanical and electrical stimulus, thereby promoting stem cells osteogenic differentiation, osteoblast mineralization, and bone metabolism homeostasis. Understanding the intricate interactions among these pathways and their regulatory mechanisms is crucial for developing advanced therapeutic strategies to promote bone repairing and treat bone metabolism disorders. Further research is needed to fully elucidate osteogenesis molecular disorders. Further research is needed to fully elucidate osteogenesis molecular mechanism and explore the potential of piezoelectric materials in bone tissue engineering.

## 4 Piezoelectric Materials for Treating Diverse Orthopedic Disorders

### 4.1 Cartilage Damage

Cartilage is a soft, flexible, sponge‐like structure that caps the ends of bones at joints. Once damaged, cartilage recovery is challenging due to its lack of true vascularization and its complex viscoelastic and anisotropic properties.^[^ ^171^ ^]^ Clinical treatments often lead to chronic pain, and pharmacological approaches typically prove ineffective. Although autologous or allogeneic bone cartilage grafts are used, they also face limitations including exceeding critical damage size, limited donor sources, and donor site trauma, making joint replacement surgery as a common solution.

**Figure 4:**
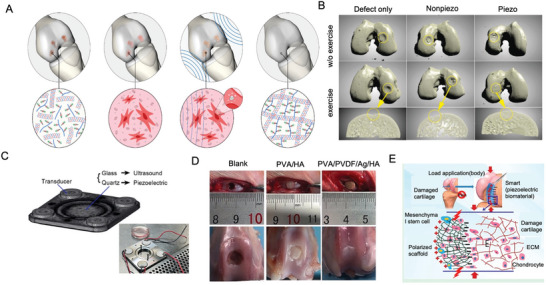
Piezoelectric materials promote cartilage repair. A) Ultrasound (US) stimulation, utilizing nanomaterials piezoelectric to stimulate cartilage tissue regeneration. Reproduced with Permission.^[^ ^172^ ^]^ Copyright 2024, American Chemical Society. B) Microscopic CT reconstruction of subchondral bone. Reproduced with Permission.^[^ ^173^ ^]^ Copyright 2022, American Association for the Advancement of Science. C) Piezoelectricity promotes chondrocyte migration. Reproduced with Permission.^[^ ^174^ ^]^ Copyright 2020, Elsevier BV. D) Rabbit osteochondral defect model. Reproduced with Permission.^[^ ^176^ ^]^ Copyright 2022, MDPI. E) Electrospinning technology simulates natural cartilage structure. Reproduced with Permission.^[^ ^177^ ^]^ Copyright 2019, American Chemical Society.

Tissue engineering scaffolds derived from chondrocytes and bone marrow stem cells have been extensively explored. The application of smart biomaterial scaffolds that respond to external physical stimuli and convert them into signals recognizable by cells represents a promising approach.^[^ ^12^ ^]^ Utilizing piezoelectric materials combined with ultrasonic stimulation represents a promising wire‐free method for inducing cartilage formation regeneration. Piezoelectric hydrogels containing BTO, when stimulated by US under precisely controlled parameters (1 MHz and 50 mW cm^−^^2^ for 5 min every 2 days over 10 days), significantly enhance the immune response in vitro and maintain the capacity to promote cartilage regeneration, even in inflammatory environments (**Figure** **4**A).^[^ ^172^ ^]^ Combining biodegradable piezoelectric tissue scaffolds with controlled mechanical activation may offer a promising approach for treatment. Biodegradable piezoelectric PLLA nanofiber scaffolds can act as battery‐free stimulators to encourage cartilage generation and regeneration under applied force or joint load. Rabbits with critical size osteochondral defects treated with piezoelectric scaffolds and exercise therapy exhibited transparent cartilage regeneration and fully healed cartilage rich in chondrocytes and type II collagen after 1 to 2 months, whereas rabbits treated with nonpiezoelectric scaffolds and exercise therapy showed unfilled defects and limited healing^[^ ^173^ ^]^ (Figure 4B).

Piezoelectric stimulation has been shown to enhance BMMSC migration, a behavior also observed in primary chondrocytes isolated from neonatal mouse articular cartilage. Inhibition of PKCγ attenuated the cellular reorganization induced by piezoelectric fields, highlighting the role of piezoelectricity in promoting various stages of fracture healing and cartilage formation^[^ ^174^ ^]^ (Figure 4C). A biomimetic electrospun PLLA nanofiber pad with gradient piezoelectric properties has also been developed to drive comprehensive osteochondral differentiation in rat MSCs.^[^ ^175^ ^]^ When exposed to an electric field, the nanofiber pad polarizes, generating gradient piezoelectric forces through linear strength variations. Contraction forces generated by cell adhesion further amplify these gradients, creating piezoelectric potentials along the scaffold that facilitate a smooth transition between cartilage and bone. This continuous gradient structure provides new insights into cartilage tissue regeneration by unifying cartilage and bone formation within a single scaffold.

Another innovative design involves a bionic hydrogel incorporating piezoelectric materials and silver nanowires, endowing it with both piezoelectric and antibacterial properties to enhance tissue regeneration^[^ ^176^ ^]^ (Figure 4D). This multifunctional hydrogel represents a significant advancement in the application of piezoelectric materials for cartilage repair, demonstrating the potential for combined therapeutic and regenerative functions.

Using electrospinning technology, poly(3‐hydroxybutyrate‐3‐hydroxyvalerate) (PHBV) doped with BaTiO_3_ was engineered to mimic the structure and piezoelectric coefficients of natural cartilage^[^ ^4^ ^]^ (Figure 4E). Research has shown that this scaffold not only enhances the proliferation, migration, and growth of chondrocytes derived from human mesenchymal stem cells but also boosts the expression of type II collagen genes. Compared with nonpolarized pure PHBV or PHBV without BaTiO_3_, this piezoelectric scaffold significantly promotes cartilage regeneration.^[^ ^4^, ^177^ ^]^

These advancements in piezoelectric materials highlight their potential to revolutionize cartilage repair and regeneration, offering new hope for treatments that are more effective and less invasive than conventional methods.

### 4.2 Hard Bone Defect

Piezoelectric materials are highly valuable in promoting osteogenesis due to their ability to generate electrical signals through mechanical motion via the piezoelectric effect, thereby avoiding the drawbacks associated with transcutaneous wires and external batteries.^[^ ^178^ ^]^ Known piezoelectric ceramic materials are effective in promoting bone repair and regeneration, and the integration of polymers can significantly enhance the biocompatibility of these ceramics.

**Figure 5:**
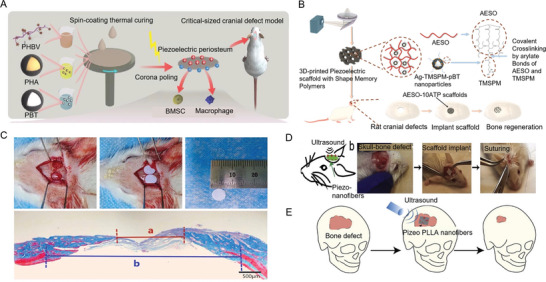
Piezoelectric materials accelerate skull regeneration. A) The preparation process of ferroelectric materials. Reproduced with Permission.^[^ ^5^ ^]^ Copyright 2023, American Chemical Society. B) Preparation process of AESO scaffold (AESO‐10ATP scaffold) doped with Ag TMSPM pBT nanoparticles (NPs) and 10% Ag TMSPM pBT NPs. Reproduced with Permission.^[^ ^179^ ^]^ Copyright 2023, Wiley. C) Preparation of a rat skull defect model and implantation of a membrane. Reproduced with Permission.^[^ ^85^ ^]^ Copyright 2022, Dove Medical Press Ltd. D) Repair of rat skull. Reproduced with Permission.^[^ ^184^ ^]^ Copyright 2020, Elsevier BV. E) Using biodegradable piezoelectric poly‐l‐lactic aicd (PLLA) nanofibers and noninvasive ultrasound (US) to generate well controlled, on‐demand, and stable surface charges for bone regeneration. Reproduced with Permission.^[^ ^184^ ^]^ Copyright 2020, Elsevier BV.

The effectiveness of treatment outcomes for bone defects varies depending on the defect size. Small defects are swiftly covered by periosteum, which facilitates the migration, proliferation, and differentiation of OBs, leading to rapid bone formation. However, defects exceeding critical size are not fully covered by the periosteum, resulting in slower healing or incomplete integration. A novel biomimetic periosteum was developed, composed of a biodegradable PHBV polymer matrix enhanced with antioxidant polydopamine‐modified hydroxyapatite (PHA) and BT. This multifunctional piezoelectric periosteum, created using the spin coating method, demonstrates excellent biocompatibility, osteogenic activity, and immunoregulatory function in vitro. In vivo experiments on intracranial defect models have shown that the biomimetic periosteum with piezoelectric stimulation not only accelerates new bone formation but also effectively inhibits intracranial inflammation and infection. This suggests a new method for rapid bone regeneration through piezoelectric stimulation^[^ ^5^ ^]^ (**Figure** **5**A).

Additionally, conductive composite materials containing glycerophosphate (GP) have been evaluated using polycaprolactone (PCL) scaffolds in male Wistar rats. These scaffolds, when implanted into a critical‐sized (5 mm × 5 mm) skull defect and stimulated with electrodes, delivered a current (10 mA for 5 min every 2 weeks), which showed that the combination of electrically active scaffolds and electrical stimulation was an effective method to enhance tissue formation and reduce osteoclast activity after 60 and 120 days.^[^ ^32^ ^]^ Furthermore, a novel piezoelectric acrylate hybrid scaffold doped with epoxidized soybean oil, Ag‐TMSPM‐pBT NPs (AESO‐ATP), was 3D printed using digital light processing. Through covalent functionalization with conductive Ag NPs and TMSPM, the piezoelectric properties of the AESO scaffolds were enhanced. The scaffold, containing 10 wt% Ag‐TMSPM‐pBT NPs, exhibited a piezoelectric coefficient (*d*_33_) of 0.9 pC N^−1^ and an output current of 146.4 nA, comparable to bone tissue. These scaffolds, with excellent shape memory function, effectively promoted osteogenic differentiation of bone marrow stromal cells in vitro and bone repair on rat skulls^[^ ^179^ ^]^ (Figure 5B).

BT/PLA composite films with varying BT ratios were prepared using the solution casting method. With a BT content of 20%, the piezoelectric coefficient (*d*_33_) reached its peak at 7.03 ± 0.26 pC N^−1^. After 12 weeks, it stabilized at 4.47 ± 0.17 pC N^−1^, aligning with the typical range of bone piezoelectric constants. Post in vitro polarization, the 20% BT group exhibited excellent adhesion and enhanced activity in MC3T3‐E1 cells. In rat skull defect sites, this group significantly accelerated bone regeneration. By 4 weeks postsurgery, considerable new bone formation was evident at the defect edges, and extensive bone marrow cavities had formed^[^ ^85^ ^]^ (Figure 5C). Similarly, BaTiO₃/multiwalled carbon nanotube (NT)/collagen (BMC) membranes with hydrophilic surfaces and improved piezoelectric properties promote bone regeneration. When combined with low‐intensity pulsed ultrasound (LIPUS), these membranes restore the natural electrical microenvironment of bone, activating Ca^2^⁺ endocytosis, and supporting cranial bone regeneration in a mouse model.^[^ ^180^ ^]^

Polyvinylidene fluoride trifluoroethylene/barium titanate ((PVDF‐TrFE)/BT) membranes have also been shown to promote bone formation in vivo. When implanted into rat skull defects, these membranes were compared with polytetrafluoroethylene (PTFE) membranes. Histomorphometric and gene expression analyses conducted at 4 and 8 weeks postimplantation revealed that P(VDF‐TrFE)/BT membranes facilitated a higher rate of bone formation than PTFE. Gene expression analyses showed enhanced osteoblast differentiation, with increased levels of RUNX2, bone sialoprotein, osteocalcin, and nuclear factor κB receptor activator ligand, as well as osteoprotegerin, suggesting that P(VDF‐TrFE)/BT membranes are more conducive to osteoblast differentiation than PTFE.^[^ ^181^ ^]^ In addition, scaffolds made from PVDF‐TrFE, fabricated via photolithography, demonstrated the ability to enhance bone regeneration by promoting preosteoblastic cell differentiation without biochemical stimulation, highlighting the synergy between surface patterns and intrinsic electroactivity.^[^ ^182^ ^]^

A biodegradable piezoelectric scaffold was created using PLLA nanofibers combined with noninvasive US stimulation, enabling remote control of electrical stimulation without the need for batteries. These biodegradable PLLA nanofiber scaffolds generate controllable surface charges under US, enhancing osteogenesis and bone regeneration.^[^ ^183^, ^184^ ^]^ The PLLA nanofibers create an environment akin to the ECM, promoting cell growth and differentiation while also providing direct electrical stimulation through remotely applied sound waves. The effectiveness of these scaffolds in enhancing osteogenic differentiation was demonstrated by measuring ALP, Alizarin red staining, and the expression of osteocalcin and osteogenic genes in vitro. Furthermore, a mouse skull defect model experiment was performed, creating a 3.5 mm bone defect in the mouse skull, followed by the placement of materials and US treatment (Figure 5D). X‐ray imaging and nuclear fast red ALP staining, along with the expression of collagen 3.6‐GFP topoaz fluorescent reporter gene and toluidine blue staining, confirmed that piezoelectric materials combined with US yielded the best therapeutic outcomes for bone defects^[^ ^183^, ^184^, ^185^ ^]^ (Figure 5E). These advancements highlight the significant potential of piezoelectric materials in promoting bone repair and regeneration, offering new and effective treatments for bone defects.

**Figure 6:**
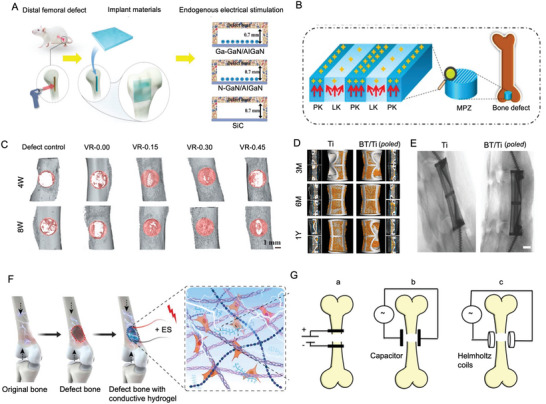
Piezoelectric materials facilitate the repair of the femur and cervical spine. A) Repair of rat femur. Reproduced with Permission.^[^ ^186^ ^]^ Copyright 2020, Wiley. B) Femoral repair materials. Reproduced with Permission.^[^ ^187^ ^]^ Copyright 2017, Ivy spring International Publisher. C) Representative three‐dimensional reconstructed and two‐dimensional sagittal‐plane micro‐CT images of bone regeneration in the rat femoral defect models at 4 and 8 weeks after implantation. Reproduced with Permission.^[^ ^188^ ^]^ Copyright 2021, Elsevier BV. D) Postoperative X‐ray film of artificial vertebral body implantation. Reproduced with Permission.^[^ ^13^ ^]^ Copyright 2023, Elsevier BV. E) Micro‐CT reconstruction of the artificial vertebral bodies and internal new bone tissues. Reproduced with Permission.^[^ ^13^ ^]^ Copyright 2023, Elsevier BV. F) Reconstruction of microenvironment for accelerated bone regeneration in bone defect areas using ES. Reproduced with Permission.^[^ ^189^ ^]^ Copyright 2024, Wiley. G) Methods of stimulating regeneration of fracture cracks. Reproduced with Permission.^[^ ^190^ ^]^ Copyright 2024, Royal Society of Chemistry.

Recently, polarity‐controlled GaN/AlGaN materials have been used to induce endogenous electrical stimulation to enhance bone regeneration (**Figure** **6**A). By manipulating the direction and amplitude of piezoelectric and spontaneous polarization within the functional layer GaN, surfaces with opposite polarity and polarization are created, generating potential within the physiological range. Ga polar GaN/AlGaN nanofilms (negatively charged surfaces) have shown faster and more effective bone healing compared to N polar GaN/AlGaN (positively charged surfaces). Additionally, the migration, recruitment, and osteogenic differentiation of bone marrow mesenchymal stem cells are greatly facilitated by the Ga polar GaN/AlGaN heterostructure.^[^ ^186^ ^]^

The microenvironment where bones are located is composed of regions of piezoelectric collagen materials and nonpiezoelectric noncollagen materials. By utilizing KNN ceramics and selective laser irradiation, a mixture of high‐voltage orthorhombic and tetragonal phases is transformed into a low‐voltage tetragonal phase (Figure 6B). This transformation creates material surfaces that simulate the bone microenvironment by mimicking electrical signals of this scale. Compared to unmodified KNN ceramics, cells cultured on KNN ceramic surfaces with modified high and low voltage regions showed enhanced expression of osteogenic differentiation markers, such as Runt‐related transcription factor 2 (Runx2) and ALP. Subsequently, a rabbit femoral condyle implantation experiment demonstrated that compared to the KNN and hydroxyapatite (HA) control group, the micro piezoelectric structures (MPZs) exhibited superior osteogenic performance.^[^ ^187^ ^]^

Bone marrow stromal stem cells are capable of differentiating into various tissue cells derived from both mesoderm and neuroectoderm, including cells typically found in muscle, liver, OBs, cartilage (chondrocytes), connective tissue (fibroblasts), and neural tissues (glial cells, neuronal cells), as well as hematopoietic stem cells and stromal cells. The use of bone marrow mesenchymal cells has been particularly effective in promoting the healing of fractures and repairing cartilage damage. An implantable self‐powered generator (ISPG) has been developed to address the challenge of energy supply for driving electronic devices and electrical stimulation therapy within the body^[^ ^188^ ^]^ (Figure 6C). These implantable devices harness biomechanical energy to provide electrical stimulation for host therapies. The interstitial fluid, cells, tissues, or organs at the target site act as electrodes and load circuits for the biological generator. This approach not only overcomes the limitations of traditional implantable generators but also expands the applicability of such devices. In experimental models with rats, this nanogenerator has shown significant promotion of bone repair in the femur, effectively balancing biocompatibility and bone repair outcomes.

Hydrothermal synthesis was employed to coat a uniform BaTiO_3_ layer on a 3D printed Ti6Al4V scaffold, creating a piezoelectric BaTiO_3_/Ti6Al4V (BT/Ti) scaffold. This scaffold exhibits piezoelectricity and has demonstrated good biocompatibility with RAW264.7 macrophages following polarization. In vitro studies showed that the piezoelectric effect of the polarized BT/Ti scaffold enhances M2 polarization of macrophages, fostering immune regulation and osteogenesis. A sheep model was used to evaluate the clinical application, where a precise total vertebral resection surgery was performed, and an artificial vertebral body from the BT/Ti (polarized) material was implanted (Figure 6D,E). The design of the artificial vertebral bodies was optimized through topology and biomechanical engineering. These bodies were secured with two locking screws, and X‐ray images taken 12 months postsurgery confirmed their correct positioning. The BT/Ti (polarized) artificial vertebral bodies effectively promoted live bone integration and repair.^[^ ^13^ ^]^

Building on these approaches, a BD‐ES system, composed of a hybrid triboelectric‐piezoelectric nanogenerator (HTP‐NG) and a conductive hydrogel, generates electrical pulses during motion to stimulate tissue regeneration. The hydrogel matrix, consisting of BP@PDA nanosheets and AlgMA, enhances electrical conductivity and provides mechanical support for tissue repair. In vitro, this system accelerated BMSC proliferation, promoted osteogenic differentiation, and stimulated angiogenesis and connective tissue development, while in vivo experiments confirmed enhanced bone regeneration and osteogenesis‐related protein expression (Figure 6F).^[^ ^189^ ^]^

Additionally, chitosan‐barium titanate (C/BT) composite membranes doped with 50% BT NPs provide a battery‐free approach to bone healing through capacitive coupling. These membranes generate electric fields at fracture sites and accelerate regeneration with excellent biocompatibility, making them a promising solution for orthopedic applications (Figure 6G).^[^ ^190^ ^]^

**Figure 7:**
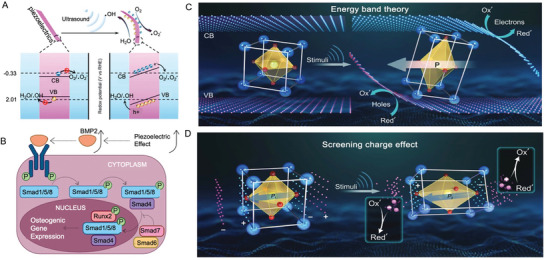
Piezocatalytic application by organic materials. A) Schematic diagram of ultrasonic induced piezoelectric polarization of piezoelectric materials, which may drive the reaction of reactive oxygen species (ROS) production on their surface. Reproduced with Permission.^[^ ^197^ ^]^ Copyright 2020, American Chemical Society. B) Schematic diagram of enhanced osteogenic mechanism related to piezoelectric effect. Reproduced with Permission.^[^ ^214^ ^]^ Copyright 2022, Elsevier BV. C,D) Piezocatalytic mechanism: Energy band theory and screening charge effect. Reproduced with Permission.^[^ ^199^ ^]^ Copyright 2023, Wiley.

Triggered by mechanical energy, piezoelectric catalytic materials can re‐release electrons or holes and catalyze the re‐oxidation of substrates, a process referred to as piezoelectric catalysis. In medical applications, these materials function as intelligent biomaterials that can respond to mechanical stimulation, influencing their interactions with cells and biological processes.^[^ ^191^, ^192^, ^193^, ^194^ ^]^ Piezoelectric catalysts can release electrons under uncontrolled stimulation to catalyze the redox reactions of substrates such as water and oxygen. This results in the production of ROS, which are utilized to kill cancer cells, degrade toxic organic compounds, or sterilize, all without the limitations of tissue penetration. This capability is particularly valuable given that electric fields can significantly influence tissue development and regeneration^[^ ^79^, ^195^, ^196^, ^197^, ^198^ ^]^ (**Figure** **7**A,B).

Utilizing the tunable electronic states of piezoelectric materials, chemical reactions can be initiated or accelerated depending on the chemical properties of the surrounding medium and the resulting strain. To date, two types of piezoelectric catalytic mechanisms have been identified: band theory and the shielding charge effect.^[^ ^199^ ^]^ According to the band theory, mechanical stimulation polarizes piezoelectric materials, leading to material deformation due to displacement of the charge center. The polarized positive and negative charges are then distributed on both sides of the material, and these generated charges participate in chemical reactions. The potential created by the piezoelectric effect sets the energy levels for the valence band (VB) and conduction band (CB) of piezoelectric materials, facilitating charge exchange at the interface of the piezoelectric material and enabling effective catalysis of redox reactions^[^ ^200^, ^201^, ^202^ ^]^ (Figure 7C). In the shielding charge effect, the piezoelectric potential acts as a drift force, catalyzing the reaction. Here, the charge involved in the redox reaction is the surface adsorption shielding charge from the external system, rather than the internal charge generated inside the material. Therefore, for a reaction to be initiated, the magnitude of the piezoelectric potential must meet or exceed the redox potential^[^ ^199^, ^200^ ^]^ (Figure 7D).

### 4.3 Osteoporosis

**Figure 8:**
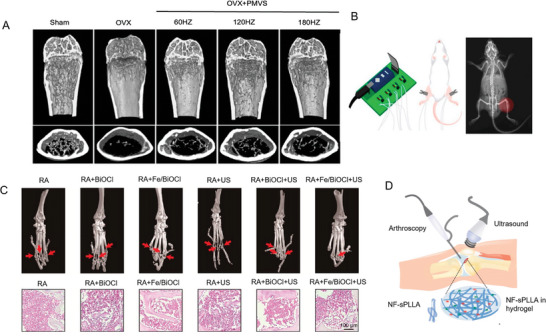
Piezoelectric materials promote the regeneration of osteoporosis, rheumatoid arthritis (RA), and osteoarthritis. A,B) Piezoelectric microvibration reduces estrogen loss induced osteoporosis. Reproduced with Permission.^[^ ^203^ ^]^ Copyright 2021, MDPI. C) Ultrasound (US) remote selected activation mitophagy for precise treatment of RA by two‐dimensional piezoelectric nanosheets. Reproduced with Permission.^[^ ^204^ ^]^ Copyright 2023, American Chemical Society. D) Injectable and biodegradable piezoelectric hydrogel for osteoarthritis treatment. Reproduced with Permission.^[^ ^205^ ^]^ Copyright 2023, Springer Nature.

Osteoporosis is a primary cause of musculoskeletal injuries in elderly patients, characterized by dysfunction in bone formation and remodeling. This dysfunction increases bone turnover, exacerbates loss of bone mass and microstructural damage, and accelerates the onset and progression of osteoporosis. Bone‐forming cells create complex mineralized microstructures and interact with OCs to maintain bone tissue homeostasis and the microenvironment. Regulating the metabolic functions of OBs and OCs to maintain bone tissue homeostasis can be beneficial in mitigating bone loss. Researchers have developed a PMVS pulse device that incorporates a microchip pulse generator, an amplifier, a pulse wave modulator, and six ceramic piezoelectric vibration transducers (**Figure** **8**A,B). The device utilized piezoelectric vibrations to stimulate bone tissue; after 48 h of low‐intensity pulsed vibration in mice, an increase in the secretion of osteoblast‐related cytokines was observed, which helped inhibit estrogen deficiency‐induced excessive bone resorption and delay the degradation of bone mass and quality. This study highlights the therapeutic potential of biophysical mechanical interventions like PMVS in preventing osteoporosis.^[^ ^203^ ^]^

### 4.4 Rheumatoid Arthritis

Rheumatoid arthritis (RA) is a common chronic autoimmune disease, characterized by persistent joint inflammation, synovial hyperplasia, and bone destruction. Although arthroscopic synovectomy is a common clinical treatment that reduces the release of inflammatory factors by removing synovial tissue, it can cause tissue damage. The development of noninvasive treatments has sparked new approaches for managing RA. Researchers have utilized two‐dimensional piezoelectric nanosheets (NSs) capable of generating electrons and holes on surfaces that remain partially unpenetrated.^[^ ^196^ ^]^ A novel therapeutic approach involves the use of US‐assisted piezoelectric BiO doped with Fe. This Fe‐doped Fe/BiOCI material significantly enhances piezoelectric properties. When stimulated by US, the Fe/BiOCI generated electrons that reduce H+ concentrations in the outer cartilage membrane, disrupting H+ supply in the cartilage matrix. This effect leads to depolarization of the MMP, triggering autophagy in mitochondrial inflammatory regions to eliminate sources of ROS production (Figure 8C). Cell and RA model experiments have shown that after treatment, inflammatory markers such as TNF‐α significantly decreased, while autophagy markers like Atg5 significantly increased, suggesting that Fe/BiOCI materials can induce mitochondrial autophagy through piezoelectric US catalysis.^[^ ^204^ ^]^

### 4.5 Osteoarthritis

Osteoarthritis affects millions globally, yet current treatments primarily focus on alleviating symptoms using painkillers or anti‐inflammatory drugs. Researchers proposed a novel biodegradable implantable piezoelectric hydrogel, composed of short electrospun L‐lactic acid nanofibers embedded within a collagen matrix (Figure 8D). This hydrogel can be injected into joints and, when activated by US, generates local electrical signals that stimulate cartilage healing. In vitro, data indicates that the ultrasonic piezoelectric gel enhances cell migration and induces stem cells to secrete TGF‐β1, thereby promoting cartilage formation. In vivo, rabbits with osteochondral critical size defects treated with this ultrasonic activated piezoelectric hydrogel showed increased subchondral bone formation, improved hyaline cartilage structure, and mechanical properties close to those of healthy natural cartilage. This piezoelectric gel not only promotes cartilage healing but also holds potential for other tissue regeneration applications.^[^ ^205^ ^]^

### 4.6 Exogenous Inflammatory Bone Disease

Osteomyelitis, a primary complication of orthopedic diseases, is notorious for its high recurrence rate.^[^ ^206^ ^]^ The site of large bone defect and much implantable bone substitutes are susceptible to bacterial adhesion and subsequent form a biofilm because of the absence of soft tissue coverage and blood supply, and exacerbate bone infection. Common treatment strategies for infectious bone defects primarily involve debridement, implant removal, and systemic inflammation treatment, considered fundamental treatment principles.^[^ ^207^ ^]^ However, due to the induced foreign body reaction, the therapeutic outcomes are often suboptimal. More critically, to facilitate the bone healing process, these implants must often be removed, leading to additional surgeries and extended hospital stays. Therefore, developing biomedical materials with robust antibacterial and osteogenic properties is crucial to enhance the repair effectiveness of bone substitutes at high risk of infection.^[^ ^208^, ^209^, ^210^ ^]^

**Figure 9:**
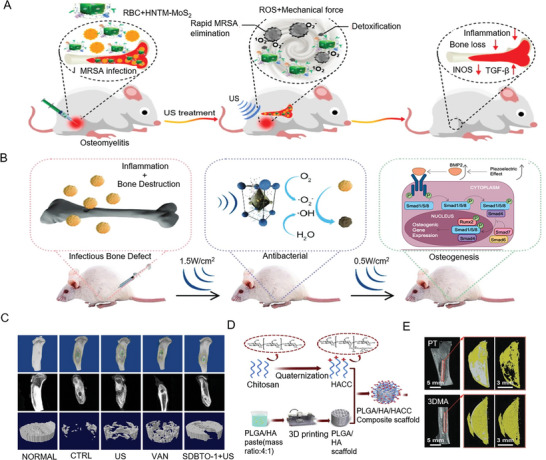
Piezoelectric materials for treating inflammatory bone diseases. A) HNTM as a sonosensitizer for the treatment of osteomyelitis. Reproduced with Permission.^[^ ^201^ ^]^ Copyright 2022, American Chemical Society. B) Adjusting ultrasound (US) piezoelectric catalysis for the treatment of bacterial infected bone defects. Reproduced with Permission.^[^ ^214^ ^]^ Copyright 2022, Elsevier BV. C) Adjusting ultrasound piezoelectric catalysis for the treatment of bacterial infected bone defects. Reproduced with Permission.^[^ ^214^ ^]^ Copyright 2022, Elsevier BV. D) 3D printed piezoelectric composite scaffold promotes bone regeneration in infectious bone defects. Reproduced with Permission.^[^ ^215^ ^]^ Copyright 2018, Elsevier BV. E) Evaluate the antibacterial, anti‐inflammatory, and osteogenic properties of 3DMA through in vivo experiments. Reproduced with Permission.^[^ ^216^ ^]^ Copyright 2023, Wiley.

Local treatment strategies have been developed. Employing HNTM as a sound sensitizer, MoS_2_ nanosheets, which exhibit a piezoelectric effect and good biocompatibility, were modified on the surface via electrostatic interaction to enhance the efficacy of sonodynamic therapy (SDT) through piezoelectric polarization and mechanical force. Additionally, HNTM‐MoS2 was coated with a layer of red blood cell (RBC) membrane to neutralize residual toxins at the infection site. This RBC‐HNTM‐MoS_2_ complex demonstrated significant efficacy in MRSA‐infected osteomyelitis models under US irradiation^[^ ^201^ ^]^ (**Figure** **9**A).

Under US irradiation, US‐responsive materials such as TiO_2_ can facilitate electron–hole pair separation and generate ROS. Recent findings indicate that piezoelectric materials such as BaTiO_3_ and ZnO can also generate ROS under US irradiation.^[^ ^211^, ^212^, ^213^ ^]^ Introducing an optimal amount of sulfur into BTO NPs enhances the asymmetry of local crystal structures in a manner akin to metal doping, directly improving the piezoelectric performance and inducing the formation of mild oxygen vacancies. This adjustment helps reduce electron–hole pair recombination, enhancing piezoelectric performance and acoustic pressure catalysis (Figure 9B). An animal model of infectious bone defect was established, and micro‐CT data was reconstructed into a 3D model to verify bone regeneration capabilities by evaluating new bone growth at the defect site. Compared with control groups, the SDBTO‐1‐AP NP+US group exhibited superior new bone growth, and the synergistic effect of piezoelectric catalysis and US effectively promoted bone repair in inflammatory bone disease defects^[^ ^214^ ^]^ (Figure 9C).

Chitosan, a naturally biodegradable and nontoxic biopolymer, has shown enhanced antibacterial performance through electrostatic binding with bacterial membranes, significantly reducing the risk of bacterial resistance. Chitosan‐coated bone scaffolds, titanium implants, and absorbable sutures have exhibited satisfactory biological properties both in vitro and in vivo. The cell compatibility and antibacterial activity of chitosan are optimized by altering the degree of substitution (DS) of quaternary ammonium. A 3D printed PLGA/HA porous scaffold grafted with chitosan was utilized, and although the progression of bone infection impacted the degradation of the PLGA/HA/chitosan composite scaffold, it demonstrated strong anti‐infection and bone repair capabilities in two different animal bone defect models with varying infections (Figure 9D). This dual‐functional new 3D printed bone scaffold holds potential application prospects for repairing infected cortical and cancellous bone defects, offering reduced risk of antibiotic resistance and satisfactory biocompatibility.^[^ ^215^ ^]^

Bacterial surfaces are typically negatively charged, primarily due to phosphate and carboxyl functional groups on the bacterial membrane. An effective method to inhibit bacterial adhesion involves altering their surface charge. Recent studies have demonstrated that negatively charged traps on surfaces can enhance antibacterial properties by reducing adhesion through electrostatic repulsion. Therefore, constructing charge traps on the surface of titanium implants represents a nontoxic, durable, cost‐effective, and straightforward antimicrobial therapeutic strategy. A three‐dimensional multifunctional architecture (3DMA) was designed on the surface of a titanium implant (Figure 9E). This architecture consisted of a graded TiO_2_ NT layer and an electrospun piezoelectric PVDF nanofiber layer. During the electrospinning process, positive charge traps were introduced into the NT layer through charge injection. The movement of bacteria through the nanofiber layer was increased through its appropriate pore size and electrostatic interaction NT layer, where bacteria were killed by positive charge traps. On the contrary, macrophages tended to adhere to the nanofiber layer. The interaction between macrophages and piezoelectric nanofibers produced self‐stimulated electric field regulating anti‐inflammatory phenotype. Lateral and three‐dimensional micro‐CT reconstructions in vivo demonstrated that the 3DMA group formed more new bone, indicating that 3DMA possesses satisfactory antibacterial, anti‐inflammatory, and bone formation‐promoting properties in vivo.^[^ ^216^ ^]^

### 4.7 Bone Tumor

Osteosarcoma, the most common malignant bone tumor, typically affects children and adolescents, occurring near the femur, tibia, or humerus. It is characterized by a high metastasis rate and poor postoperative recovery. Large bone defects and residual tumor cells after surgery or radiotherapy can impede bone healing and increase the risk of recurrence. Therefore, effective methods to eliminate residual tumors and promote bone regeneration are critical.

**Figure 10:**
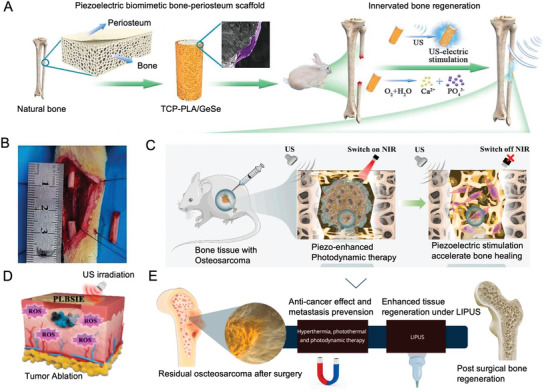
Multifunctional piezoelectric materials for osteosarcoma treatment. A) Piezoelectric biomimetic bone‐periosteum scaffold (TCP‐PLA/GeSe) enhancing innervated bone regeneration. Reproduced with Permission.^[^ ^217^ ^]^ Copyright 2024, BioMed Central. B) In vivo placement of piezoelectric scaffolds for osteosarcoma therapy and bone defect repair. Reproduced with Permission.^[^ ^217^ ^]^ Copyright 2024, BioMed Central. C) BST nanoheterostructures with near‐infrared (NIR) and ultrasound (US) activation for photodynamic therapy and accelerated bone healing. Reproduced with Permission.^[^ ^218^ ^]^ Copyright 2024, KeAi Publishing. D) Piezoelectric elastomers (PLBSIE) elastomers generating reactive oxygen species (ROS) for tumor ablation and promoting wound healing. Reproduced with Permission.^[^ ^98^ ^]^ Copyright 2023, American Chemical Society. E) Piezoelectric magnetic nanoparticles (PMNPs) for multimodal therapy (magnetothermal, photothermal, and photodynamic) and postsurgical bone regeneration. Reproduced with Permission.^[^ ^219^ ^]^ Copyright 2022, Elsevier BV.

Recent studies have introduced piezoelectric biomimetic scaffolds like the TCP‐PLA/GeSe nanofiber membrane (**Figure** **10**A). Developed using electrospinning, these scaffolds enhance innervated bone regeneration and enable photothermal ion therapy for osteosarcoma. US stimulation, they promote neurogenic differentiation by activating the calcium signaling pathway, PI3K‐AKT, and Ras signaling, while also facilitating osteogenic differentiation in BMSCs. Additionally, the scaffold's photothermal ablation capability and selenium release enhance the treatment of osteosarcoma^[^ ^217^ ^]^ (Figure 10B). Conventional photosensitizers often struggle with both treating osteosarcoma and promoting bone regeneration. To address this, BST nanoheterostructures embedded in a biopolymer hydrogel respond to tumor microenvironments (pH < 6.7) and release ROS upon exposure to US and near‐infrared (NIR) light (Figure 10C). When NIR is turned off, the remaining BST NPs were activated by US to generate piezoelectric stimulation, activating the PI3K/AKT pathway to accelerate osteogenic differentiation and bone healing.^[^ ^218^ ^]^ Piezoelectric elastomers (PLBSIE) (Figure 10D) offer another promising approach by serving as a unified platform for tumor treatment and wound healing. Upon US activation, PLBSIE generates ROS to kill tumor cells and bacteria, while the piezoelectric effect promotes cell migration and differentiation, accelerating wound repair and bone regeneration after osteosarcoma surgery.^[^ ^98^ ^]^ PMNPs (piezoelectric magnetic nanoparticles), combining barium titanate with superparamagnetic iron oxide, provide a multifunctional therapeutic platform^[^ ^219^ ^]^ (Figure 10E). These NPs offer magnetothermal, photothermal, and photodynamic effects to eradicate residual tumor cells and, under FDA‐approved LIPUS, enhance bone tissue regeneration and repair large defects from tumor resection. PMNPs also hold potential as CT contrast agents. Lastly, KNN synergizes US‐triggered piezoelectric catalysis with chemotherapy to generate ROS, significantly inhibiting tumor growth. KNN therapy promotes apoptosis and autophagy in osteosarcoma cells while exhibiting high biocompatibility, with no toxic effects observed in healthy tissues or organs during in vivo studies.^[^ ^220^ ^]^

## 5 Advanced Piezoelectric Devices in Orthopedic Medicine

Piezoelectric devices are favored for their compact size, high output voltage, mechanical simplicity, high sensitivity to applied strains, and high power density.^[^ ^221^ ^]^ Depending on their piezoelectric properties, flexibility, biocompatibility, degradability, toxicity, and side effects, implantable and wearable piezoelectric devices have been widely applied in orthopedic medicine.

### 5.1 Implantable Piezoelectric Devices in Bone Repair

#### 5.1.1 Piezoelectric Nanofiber Scaffold

**Figure 11:**
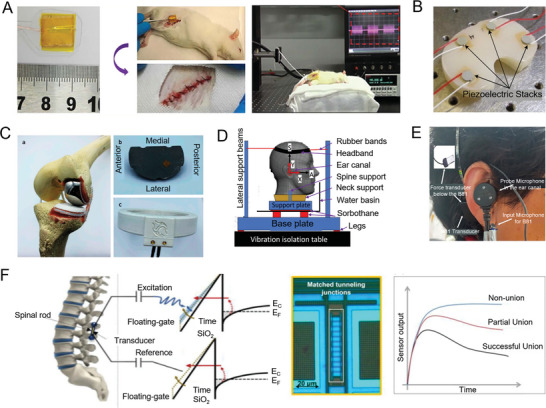
Implantable piezoelectric sensors in bone repair. A) Implantation of piezoelectric scaffolds in rat thighs to capture sensing signals during traction. Reproduced with Permission.^[^ ^223^ ^]^ Copyright 2019, MDPI. B) Assembly of an instrumented knee joint bearing equipped with four piezoelectric stacks. Reproduced with Permission.^[^ ^229^ ^]^ Copyright 2018, IOP Publishing Ltd. C) Wireless battery‐free knee implant monitoring. Reproduced with Permission.^[^ ^230^ ^]^ Copyright 2024, MDPI. D) Piezoelectric subcutaneous bone conduction (BC) device. Reproduced with Permission.^[^ ^233^ ^]^ Copyright 2018, Elsevier BV. E) Piezoelectric thin film bone conduction. Reproduced with Permission.^[^ ^238^ ^]^ Copyright 2022, Frontiers. F) Integration of a piezoelectric transducer with a spinal fusion fixation device for monitoring spinal fusion progress. Reproduced with Permission.^[^ ^241^ ^]^ Copyright 2022, IEEE Computer Society.

The piezoelectric properties of PVDF‐TrFE can vary significantly with spinning parameters, affecting its piezoelectric coefficients. For instance, the d_31_ coefficient for nanoelectrospinning can reach 16.17 pC N^−1^. Fibroblasts stimulated by the surface charge of these nanofiber scaffolds can experience a 1.6‐fold increase in their proliferation rate. These scaffolds also demonstrate good cell compatibility (**Figure** **11**A). In vivo experiments have confirmed the stable output of current and voltage during intermittent stretching, suggesting significant potential for applications in tissue engineering and bone regeneration.^[^ ^222^, ^223^ ^]^

#### 5.1.2 Piezoelectric Joint System

TKR is a prevalent treatment for injured patients, where postoperative joint failure is mainly attributed to joint loosening and instability.^[^ ^224^, ^225^, ^226^ ^]^ There remains a need to develop more objective and recognized evaluation methods. Previously, an intelligent remote monitoring system was proposed for the knee joint, primarily used to monitor the relative flexion between the femur and tibia.^[^ ^227^ ^]^ The data can be remotely transmitted to a clinical server to evaluate the progress and regression of the knee joint based on the achievable degree of flexion. However, this system does not provide in vivo data. It is feasible to use piezoelectric materials implanted in TKR devices to generate electrical energy as sensors.^[^ ^228^ ^]^ In 2018, an embedded piezoelectric transducer design was proposed (Figure 11B), placing transducers at four positions within the joint. These transducers, located in each compartment of the UHMW insert (two PZT transducers per compartment), apply force through the femoral component, providing self‐powered sensing capabilities from the bearing and allowing daily transmission of data to monitor implant loosening, wear, and fracture.^[^ ^229^ ^]^ A wireless, battery‐free smart implant system incorporating piezoelectric sensors and inductive coils was also developed to measure fixation at the implant‐cement interface of the tibial component using passive telemetry. In partial knee replacement (PKR) and TKR systems, the sensors were fixed to the upper surface of the tibial prosthesis. Fixation was measured through a pulse‐echo response induced by a triple‐coil inductive link, with minimal interference from external reader position, soft tissue, or femoral components^[^ ^230^ ^]^ (Figure 11C). Further studies evaluated the ability of this design to harvest energy from normal knee joint function.^[^ ^223^, ^229^, ^231^, ^232^ ^]^

#### 5.1.3 Piezoelectric Subcutaneous Bone Conduction

Bone conduction (BC) involves transmitting sound waves or vibrations from the air through the mechanical vibrations of the head and body structures to the inner ear, serving as an alternative to air conduction (AC) and stimulating the cochlea to generate sound perception^[^ ^233^, ^234^, ^235^, ^236^ ^]^ (Figure 11D). Clinically, BC hearing aids are particularly beneficial for patients with unilateral deafness and conductive hearing loss who cannot be aided by traditional hearing aids.^[^ ^234^, ^237^ ^]^ A novel piezoelectric transducer‐based actuator (PZTA) was tested at the conventional BAHA position on embalmed cadaver skulls, directly behind the ear. The sound transmission efficiency of this new subcutaneous BC device was quantified, confirming effective sound transmission at various positions and angles.^[^ ^233^ ^]^ A piezoelectric thin‐film force sensor was also developed to measure the force levels at the stimulus location, offering precise control. In tests with 20 hearing‐impaired subjects, the force sensor method and the ear canal method provided comparable audibility results, both significantly outperforming the traditional artificial mastoid method^[^ ^238^ ^]^ (Figure 11E). Additionally, to reduce the risk of surgical failures, a piezoelectric device was developed to assess the vibrations of the ossicular chain during middle ear surgery. Resembling a pen, this device comprises a reusable body and a disposable sensitive head featuring a piezoelectric polymer sensor, capable of recording the responses of both normal and reconstructed ossicular chains to acoustic stimuli.^[^ ^185^, ^239^ ^]^

#### 5.1.4 Spinal Fusion Surgery

Spinal fusion surgery, aimed at treating conditions such as degenerative intervertebral discs, scoliosis, trauma, and tumors, carries a significant risk of postoperative complications, affecting approximately 30% of patients.^[^ ^240^ ^]^ The successful outcome of spinal fusion largely depends on accurately evaluating its stability. While CT scans provide more detailed progress assessments compared to X‐rays, they often yield ambiguous results. Implantable spinal fusion sensors, powered by piezoelectric transducers, have been utilized to monitor the progress of spinal fusion. These sensors convert mechanical strains into electrical charges stored in volatile memory. A novel method employing a self‐powered system for monitoring lumbar fusion healing has been introduced. The system, powered by strain from spinal fixation devices, continuously senses and records changes in the elastic modulus of the fused spinal section as it increases, thereby decreasing strain levels on the fixation device (Figure 11F). This proposed system's outputs map the time evolution of each stage in the fusion process, as demonstrated in a feasibility study using a vertebral resection model. This model simulated the spinal fusion process by inserting materials with progressively increasing elastic moduli into the gap between two synthetic vertebrae.^[^ ^241^ ^]^

#### 5.1.5 Fracture Healing

Fractures, whether resulting from falls, sports injuries, or other causes, often require the surgical implantation of metal orthopedic plates or rods to stabilize the bone during healing. Accurately monitoring the healing progress and determining the appropriate time for patients to resume activities is crucial. In one innovative approach, two PZTs were embedded within standard orthopedic screws and positioned on either side of a simulated fracture—one acting as a transmitter and the other as a receiver. This setup effectively detected significant differences between intact and fractured bone sections, providing valuable data on the degree of fracture healing suitable for clinical use.^[^ ^242^ ^]^

### 5.2 Wearable Piezoelectric Devices in Sports Medicine

Traditional centralized medical services often require hospital visits, which may not adequately monitor real‐time data or address some patient needs. Wearable health monitoring devices have gained substantial attentions for their ability to detect and monitor users' movement and physiological signals, enabling timely medical intervention and necessary treatment.^[^ ^243^, ^244^, ^245^, ^246^ ^]^ Wearable or connectable health monitoring systems are considered the next generation of personal portable devices for remote medical practices.^[^ ^247^, ^248^ ^]^ Piezoelectric sensing devices allow these systems to track physiological signals in real time, offering a convenient and noninvasive method for disease diagnosis and evaluation.^[^ ^249^, ^250^, ^251^, ^252^ ^]^

#### 5.2.1 Small Motion Monitoring

**Figure 12:**
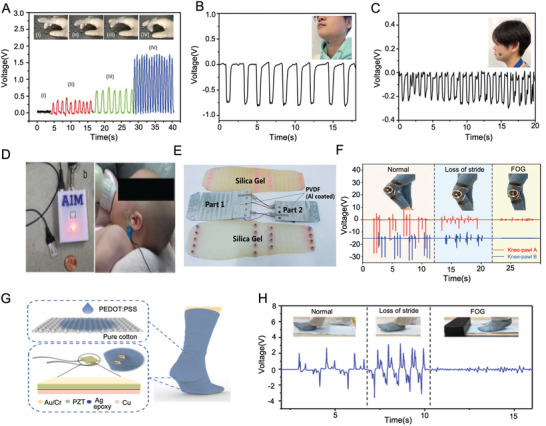
Wearable piezoelectric sensors in sports medicine. A–C) Wearable piezoelectric sensors applied to monitor various finger joint motion and swallowing and chewing activities. Reproduced with Permission. Copyright 2018, MDPI. D) Infants milk consumption. Reproduced with Permission.^[^ ^257^ ^]^ Copyright 2015, Hindawi Limited. E) Plantar pressure. Reproduced with Permission.^[^ ^258^ ^]^ Copyright 2016, MDPI. F) knee joint motion, Reproduced with Permission.^[^ ^259^ ^]^ Copyright 2021, Wiley. G,H) Gait during forward, left, and right walking. Reproduced with Permission.^[^ ^261^ ^]^ Copyright 2019, American Chemical Society.

Commonly used piezoelectric sensors in human health monitoring excel due to their outstanding mechanical performance and high sensitivity. Through an electrospinning process, P(VDF‐TrFE)/multiwalled carbon nanotube (MWCNT) composite materials are produced. After undergoing mechanical stretching treatment, these composite films exhibit a high β‐phase content, high sensitivity, and good mechanical robustness.^[^ ^253^ ^]^ When placed on the steering knuckle, the output voltage of the sensor responds proportionally to the degree of finger bending. The sensor, fixed on the wrist, can differentiate wrist movements such as upward bending, downward bending, and twisting (**Figure** **12**A). The piezoelectric pressure sensor also used for monitoring vital signs and muscle motion (Figure 12B,C). These findings suggest that P(VDF‐TrFE)/MWCNT composite film‐based piezoelectric pressure devices, which are both tensile and wearable, can produce varied responses corresponding to different types of human motion. NiO@SiO_2_/PVDF nanocomposites, characterized by excellent flexibility, light weight, and low cost, have shown distinct and easily recognizable signal patterns when placed on different fingers, which could be beneficial for monitoring the health of bedridden patients.^[^ ^254^ ^]^ These results demonstrate the potential applications of enhanced piezoelectric pressure sensors in personal healthcare and disease diagnosis. A flexible pulse sensor, capable of detecting subtle skin surface deformations caused by arterial pulses, is crucial for developing noninvasive, continuous pulse waveform monitoring systems that provide important health status parameters.^[^ ^255^, ^256^ ^]^ Unlike traditional active pulse sensors, piezoelectric pulse sensors (PPS) offer higher sensitivity, stability, and lower power consumption, representing a promising solution for flexible pulse sensors. This study introduced a highly sensitive and flexible PPS for detecting surface deflections at the micrometer level using single‐crystal Group III nitride thin films. The biocompatible flexible PPS effectively detects detailed characteristic pulse waveforms from most arterial pulse sites when attached to the skin surface without external pressure. A flexible PPS with a simple sandwich structure was developed using single‐crystal III‐N thin films through a thin film layer transfer method. Its sensitivity to small‐scale forces and deflections was systematically studied, and its durability evaluated through experiments. The sensor's ability to display different pulse intensities at various arterial pulse locations also allowed for the extraction of useful physiological information, such as arterial augmentation index (AIx) and pulse wave velocity (PWV). Additionally, it demonstrated the capacity to detect changes in pulse waveforms due to exercise and deep breathing, enabling continuous monitoring of arterial pulses.^[^ ^244^ ^]^

#### 5.2.2 Infant Feeding Behavior Monitoring

Infant feeding behavior is crucial for understanding the development of conditions such as obesity, yet current monitoring methods are limited and yield minimal quantitative data. A study utilized a piezoelectric mandibular motion sensor to monitor the jaw movements of 10 infants during feeding sessions^[^ ^257^ ^]^ (Figure 12D). The sensor, attached directly below the baby's ear and behind the lower jaw, coupled with a camera setup, recorded oral and chin movements before and during feeding. This approach enabled the accurate counting of sucking instances, demonstrating the feasibility of piezoelectric pressure sensors for monitoring mandibular movements.

#### 5.2.3 Gait Monitoring

Gait analysis plays a vital role in managing chronic diseases such as stroke, Parkinson's disease, and diabetes. With advancements in the Internet of Things (IoT), remote, accurate, and continuous gait monitoring technologies have emerged, reducing logistical burdens on patients and improving rehabilitation outcomes. Key parameters such as speed, stride length, and plantar stress distribution are critical for assessing disease progression and guiding recovery strategies. Sandwich insoles with piezoelectric sheets have been designed with corrugated ribs on the upper plates and grooves on the lower plates. These insoles contain multiple PVDF film sheets fixed at the ends of the plates; when the upper plate is pressurized, the PVDF film stretches along the 1‐axis, generating electrical charges that are collected by electrodes^[^ ^258^ ^]^ (Figure 12E). Additionally, 3D printing and piezoelectric generators have been used to convert complex three‐dimensional lower limb movements into linear motions, which are analyzed using the MC‐EH‐HL system for energy harvesting and monitoring lower limb activity^[^ ^259^ ^]^ (Figure 12F). A team also developed PVDF‐based insole sensors with 24 sensing points to measure vertical foot pressure, ensuring high two‐dimensional spatial resolution and flexibility. These sensors incorporate wireless communication capabilities to transmit data to remote terminals, further enhancing monitoring efficiency.^[^ ^260^ ^]^ Furthermore, a self‐powered sock has been developed, employing a piezoelectric sensor coated with lead PZT based on poly(3,4‐ethylenedioxythiophene) polystyrene sulfonate (PEDOT:PSS) (Figure 12G). This sensor not only evaluates sweat levels but also directly outputs mechanical contact signals, transforming traditional socks into energy‐harvesting devices that facilitate both load sensing and energy waste reduction, showcasing the synergy between wearable technology and monitoring devices^[^ ^261^ ^]^ (Figure 12H). Additionally, most motion recognition technologies achieve accurate sensing through tight‐fitting suits, whereas more flexible piezoelectric sensors are now designed for loose clothing. These sensors, attached to the knees and buttocks of loose trousers, effectively monitor walking, standing, and movements around beds and wheelchairs, enhancing the monitoring capabilities for the lower body.^[^ ^262^ ^]^ An innovative piezoelectric heterostructure has been designed using magnetic springs to harness energy from human foot impacts, suitable for low‐frequency (e.g., less than 2 Hz) and high‐amplitude motions. This technology protects the piezoelectric single‐crystal cantilever with adjustable maximum tip displacement to accommodate various impact loads. Experimental validation showed that as walking or running speed increased from 3 to 7 km h^−1^, the RMS voltage of the device monotonically increased. Similarly, the maximum average power output also rose with the increase of initial separation distance in the magnetic springs, suggesting that further optimization of piezoelectric heterostructures could enhance their efficiency and application scope.^[^ ^263^ ^]^

#### 5.2.4 Bone Density Detection

The prevalence of osteoporosis among the elderly, primarily caused by the loss of bone calcium, necessitates regular monitoring of bone health. Traditional bone density testing equipment, while effective, is often not conducive to frequent or real‐time monitoring.^[^ ^264^ ^]^ To address this challenge, wearable real‐time medical devices have emerged as a significant area of research. Leveraging the principles of quantitative US, researchers have developed a high‐performance, flexible bone US detection system utilizing axial transmission technology. This innovative system uses a novel rare earth‐doped PMN‐PZT piezoelectric ceramic, synthesized through a solid‐state reaction. The material's properties were thoroughly characterized using X‐ray diffraction and scanning electron microscopy, ensuring the sensor's effectiveness and reliability for clinical applications.^[^ ^265^ ^]^

## 6 Outlook

### 6.1 Limitations and Challenges

Despite the promising potential of piezoelectric materials in orthopedic medicine, several challenges—related to long‐term stability, biocompatibility, toxicity, and mechanical durability—must be addressed to ensure their safe and effective clinical use.

#### 6.1.1 Long‐Term Stability

The performance of piezoelectric materials, especially under physiological conditions, tends to degrade over time. Environmental factors such as moisture, mechanical stress, and temperature can affect material properties. Organic piezoelectric polymers, such as PVDF, are likely to have dipole relaxation, causing a gradual decrease in piezoelectric output over time.^[^ ^44^ ^]^ Additionally, the exposure to body fluids, which increases material conductivity, can suppress electrical performance by disrupting the asymmetrical dipole alignment that is critical to piezoelectric performance.^[^ ^266^ ^]^ This challenge necessitates the development of materials with stable piezoelectric properties, even in wet environments, or protective encapsulation strategies for implantable devices.

#### 6.1.2 Biocompatibility Issues

Biocompatibility is essential for long‐term biomedical applications. Inorganic piezoelectric materials such as lead PZT offer high performance but pose concerns due to poor biocompatibility and mechanical brittleness, which limit their direct use in implants.^[^ ^44^ ^]^ Organic materials such as PLLA and PVDF are more compatible with biological tissues, but they exhibit lower piezoelectric coefficients, limiting their therapeutic performance.^[^ ^267^ ^]^ Hybrid composite materials that combine the advantages of inorganic and organic components have shown promise, but achieving seamless integration with biological system remains a challenge.

#### 6.1.3 Potential Toxicity

One of the most pressing concerns is the toxicity of materials containing harmful elements like lead. Lead‐based piezoelectric ceramics, such as PZT, can release toxic ions, leading to environmental and health risks, especially for long‐term implants.^[^ ^44^ ^]^ Lead‐free alternatives, such as BaTiO_3_ and potassium sodium niobate KNN, exhibit reduced toxicity but still lag behind PZT in terms of piezoelectric performance.^[^ ^268^ ^]^ Additionally, potential leaching of NPs from piezoelectric composites raises safety concerns, necessitating in‐depth studies on degradation products and their effects on biological systems.

#### 6.1.4 Mechanical Fatigue and Structural Integrity

Inorganic piezoelectric materials, although highly efficient, tend to be brittle, making them susceptible to mechanical fatigue and fracture under continuous deformation.^[^ ^60^ ^]^ For orthopedic applications, materials must maintain both mechanical and electrical properties under prolonged loading. Organic piezoelectric polymers, while more flexible, exhibit lower mechanical strength and piezoelectric output, requiring strategies to improve their toughness through reinforcement with inorganic nanofillers or by optimizing their crystalline structure. Further research is needed to design fatigue‐resistant materials capable of withstanding cyclic mechanical loading that is typical for orthopedic implants.

#### 6.1.5 Fabrication Challenges and Clinical Translation

The integration of piezoelectric materials with host tissues, such as bone or cartilage, poses technical challenges. For example, achieving stable mechanical‐electrical coupling without compromising tissue function is complex. Moreover, processing techniques such as electrospinning or 3D printing must be optimized to fabricate flexible and biocompatible piezoelectric scaffolds with consistent performance. Regulatory approval processes also introduce hurdles, as piezoelectric implants must undergo rigorous safety and efficacy testing before they can be approved for clinical use.

#### 6.1.6 Future Directions and Insights

While piezoelectric materials and devices advance rapidly in the medical field, substantial studies have demonstrated their significant potential in orthopedic medicine, particularly bone repair and regeneration. This paper reviews piezoelectric materials and principles and their innovative applications in the diagnosis and treatment of orthopedic diseases through enhancing bone tissue regeneration, alleviating inflammation, and monitoring bone health. These studies revealed the diversity and wide implementations of piezoelectric materials in orthopedics through sensing and therapeutizing. In recent years, flexible electronics and advanced manufacturing technologies have facilitated the development of wearable and implantable piezoelectric systems, which might further pave the way for personalized medicine and precision therapy.

**Figure 13:**
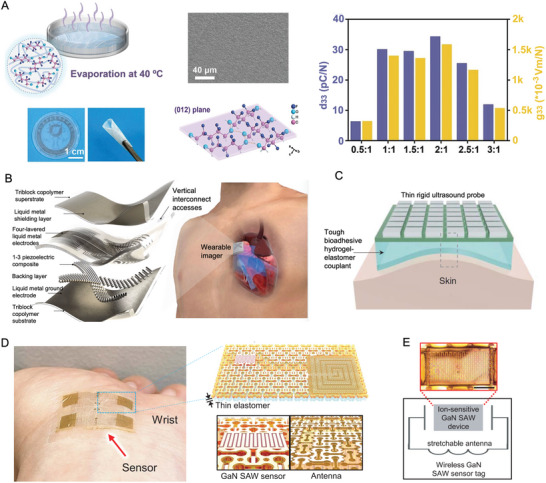
Potential piezoelectric systems in orthopedic medicine. A) High‐efficiency piezoelectric materials prepared by low‐temperature evaporation. Reproduced with Permission.^[^ ^269^ ^]^ Copyright 2024, American Association for the Advancement of Science. B,C) Wearable ultrasound (US) imaging device for real‐time monitoring of blood flow and tissue changes. Reproduced with Permission.^[^ ^270^ ^]^ Copyright 2023, Nature Publishing Group. D,E) GaN surface acoustic wave (SAW) sensors and skin sensors for postoperative biochemical environment monitoring. Reproduced with Permission.^[^ ^271^ ^]^ Copyright 2022, American Association for the Advancement of Science.

**Figure** **13** illustrates some of the latest applications of piezoelectric technology in other medical disciplines, which might provide more sights for future piezoelectric orthopedic applications. Figure 13A illustrates a high‐efficiency piezoelectric material prepared by low‐temperature evaporation at 40 °C.^[^ ^269^ ^]^ This material can be utilized to develop novel sensors and energy harvesters for monitoring biomechanical changes in bone health and repair processes, providing real‐time data support. Figure 13B,C depicts a wearable imaging device that uses liquid metal and vertical interconnect structures to achieve real‐time monitoring of the heart.^[^ ^270^ ^]^ The device adheres closely to the skin, capturing dynamic images and electrical signals of the heart. In orthopedic medicine, such wearable devices can be used to monitor blood flow and tissue changes during fracture healing, providing data support for personalized treatment and improving rehabilitation efficiency. Figure 13D,E demonstrates a skin sensor integrated with a GaN surface acoustic wave (SAW) sensor.^[^ ^271^ ^]^ This system includes a wireless GaN SAW sensor tag and a stretchable antenna for monitoring ion concentration and environmental changes. It can be used to monitor the biochemical environment postorthopedic surgery, such as detecting inflammatory markers or drug release, ensuring the safety and effectiveness of the recovery process.

These advanced technologies demonstrate the significant potential of electrical stimulation therapy, piezoelectric materials, and wearable monitoring devices in orthopedic medicine. Through real‐time monitoring and regulation, they can significantly enhance treatment outcomes and patient rehabilitation quality. The interdisciplinary application of these technologies indicates a future trend of integration between orthopedic medicine, materials science, and electronic engineering.

#### 6.1.7 Rational Design of Piezoelectric Nanomaterials

The future research direction involves in‐depth studies on the electromechanical performance of piezoelectric nanomaterials under ultrasonic stimulation to design more efficient piezoelectric therapeutic schemes. Understanding the response behavior of nanomaterials at different US power and frequency settings is crucial for optimizing therapeutic effects. Currently, the selection of nanomaterials and US parameters is largely empirical, and the response of piezoelectric nanomaterials under US is difficult to accurately characterize. Future research should focus on specific piezoelectric therapy application scenarios, systematically designing the types, structures, and US conditions of nanomaterials to achieve rational design of piezoelectric therapeutic schemes. This includes using advanced simulation and experimental techniques to comprehensively characterize the behavior of nanomaterials under US and developing corresponding optimization algorithms to achieve efficient and precise therapeutic effects.

#### 6.1.8 Immunomodulatory Mechanisms

The future research direction is to explore the role of piezoelectric materials in immunomodulation, understanding how they influence inflammatory responses and immune cell behavior in bone repair. Current research primarily focuses on the direct mechanical effects of piezoelectric materials, with relatively less attention to their immunomodulatory functions. Future research should focus on the immunomodulatory roles of piezoelectric materials in bone repair processes, particularly how they regulate immune cell behavior and inflammatory responses to promote bone regeneration. By combining immunology and materials science research, developing piezoelectric materials with immunomodulatory functions could provide new strategies for comprehensive treatment of orthopedic diseases.

#### 6.1.9 Piezoelectric Biomolecules for Targeted Therapy

The future research direction is the development of smart biomaterials that can simultaneously promote bone regeneration and inhibit bone diseases (such as bone tumors and infections), significantly enhancing the comprehensive treatment of orthopedic conditions. Current smart biomaterials need further research regarding their multifunctionality, controllability, and stability in vivo. Future research can further explore the applications of smart biomaterials in targeted therapy, particularly by combining piezoelectric materials with other functional materials to develop composite materials that respond to specific physiological and pathological signals. These materials need to possess excellent biocompatibility and controlled release characteristics in vivo to achieve precise therapeutic effects.

#### 6.1.10 Miniaturized Wearable and Implantable Bone Sensors

The future research direction is to integrate piezoelectric materials into wearable and implantable sensors to achieve continuous monitoring of bone health and personalized medical care, improving diagnostic and therapeutic accuracy. Current sensors face issues with data transmission stability, power management, and long‐term biocompatibility. Future research should focus on enhancing the accuracy and usability of these sensors to ensure their widespread clinical application. Improving data transmission technology and power management systems will ensure the stability and reliability of sensors in long‐term use. Additionally, developing new piezoelectric materials with high sensitivity and low power consumption can further enhance sensor performance.

#### 6.1.11 Noninvasive Therapeutic Interventions

The future research direction is the application prospects of piezoelectric materials in noninvasive therapy. Using US‐responsive piezoelectric scaffolds can generate localized electrical stimulation to enhance bone regeneration and reduce inflammation. However, noninvasive therapeutic technologies may face challenges in controlling the intensity and precision of electrical stimulation in practical applications. Future research should continue to develop these noninvasive therapeutic technologies to provide less invasive and more effective treatment options for orthopedic patients. By optimizing US parameters and piezoelectric material performance, precise control of electrical stimulation intensity and localization can be achieved, improving therapeutic accuracy and effectiveness. Additionally, clinical trial data should be used to continuously adjust and refine therapeutic schemes for optimal outcomes.

#### 6.1.12 Integration with Advanced Manufacturing Technologies

The future research direction is to use advanced manufacturing technologies such as 3D printing to precisely control the microstructure and properties of piezoelectric scaffolds, enabling personalized therapeutic schemes and improving treatment outcomes. However, the application of 3D printing technology in the manufacturing of biological materials faces challenges such as material selection, printing precision, and cost control. Future research should focus on developing personalized therapeutic schemes based on the specific needs of individual patients, customizing scaffolds to enhance treatment outcomes and reduce complications. By continuously improving 3D printing technology, increasing material diversity and printing precision, and reducing manufacturing costs, personalized therapeutic schemes can become more widespread and feasible.

Despite significant advancements, considerable progress is still needed before piezoelectric materials can be clinically applied in orthopedics. Key areas requiring further exploration include long‐term in vivo biocompatibility and stability, tailoring piezoelectric performance to meet diverse clinical needs, and scaling up materials and devices through manufacturing for practical applications. To overcome these hurdles and unlock the full potential of piezoelectric materials in orthopedic medicine, continued interdisciplinary research integrating materials science, biomedical engineering, and clinical translation is essential. Overall, the evolution of piezoelectric technology not only offers new possibilities for bone repair and regeneration but also marks the beginning of an era of intelligent and responsive medicine.

## Conflict of Interest

The authors declare no conflict of interest.
